# Attitude Trajectory Optimization to Ensure Balance Hexapod Locomotion

**DOI:** 10.3390/s20216295

**Published:** 2020-11-05

**Authors:** Chen Chen, Wei Guo, Pengfei Wang, Lining Sun, Fusheng Zha, Junyi Shi, Mantian Li

**Affiliations:** 1 State Key Laboratory of Robotics and System, Harbin Institute of Technology, Harbin 150001, China; danny_cc@hit.edu.cn (C.C.); wguo01@hit.edu.cn (W.G.); lnsun@hit.edu.cn (L.S.); zhafusheng@hit.edu.cn (F.Z.); junyishi@hit.edu.cn (J.S.); limt@hit.edu.cn (M.L.); 2 Shenzhen Academy of Aerospace Technology, Shenzhen 518057, China

**Keywords:** attitude trajectory optimization, balance motion control, attitude fluctuation counteraction, large-size hexapod robot, control system design

## Abstract

This paper proposes a simple attitude trajectory optimization method to enhance the walking balance of a large-size hexapod robot. To achieve balance motion control of a large-size hexapod robot on different outdoor terrains, we planned the balance attitude trajectories of the robot during walking and introduced how leg trajectories are generated based on the planned attitude trajectories. While planning the attitude trajectories, high order polynomial interpolation was employed with attitude fluctuation counteraction considered. Constraints that the planned attitude trajectories must satisfy during walking were well-considered. The trajectory of the swing leg was well designed with the terrain attitude considered to improve the environmental adaptability of the robot during the attitude adjustment process, and the trajectory of the support leg was automatically generated to satisfy the demand of the balance attitude trajectories planned. Comparative experiments of the real large-size hexapod robot walking on different terrains were carried out to validate the effectiveness and applicability of the attitude trajectory optimization method proposed, which demonstrated that, compared with the currently developed balance motion controllers, the attitude trajectory optimization method proposed can simplify the control system design and improve the walking balance of a hexapod robot.

## 1. Introduction

For centuries, different mobile robots and control methods have been developed to satisfy different mission objectives with the purpose of improving the quality of human life [1,2,3]. Unlike some conventional wheeled robots which operate in structured environments, legged robots with different leg structures, such as wheel-legged types [4,5] and mammalian types [6,7,8], can perform more complex motion behaviors to fulfill tough objectives in unstructured environments, such as disaster search and rescue operations, and load-carrying transportations on rugged terrains. Due to the good environmental adaptability of legged robots, motion control of legged robots arouses the interests of many scholars. Among all the research fields of legged robot control, balance motion control is one of the most important topics for scholars to conquer, especially for large-size legged robots [9,10,11]. For legged robots, the rugged external terrain conditions, and the complex interaction between robot leg and environment together challenge the walking balance which is of crucial importance in ensuring a safe motion process. Compared with small-size legged robots, a secure walking process is the first priority which needs to be ensured for a large-size legged robot. A large-size legged robot has severe difficulties in recovering walking balance due to very large inertia and mass characteristics. In addition, large contact impacts are usually found between the feet and the terrain for a large-size legged robot. All these typical characteristics of a large-size legged robot will lead to an unbalance motion process, which may further lead to walking stability loss and cause irreversible damage to the robot components.

The walking balance performance of a legged robot is usually evaluated by the fluctuations of the body attitude angles, especially the pitch and roll angles. The attitude angles of a legged robot are continuously influenced by many factors during walking, such as the contact foot forces, the terrain conditions, etc. Based on the studies of the impact factors and inspired by the currently developed control theories, various control methods have been carried out by scholars from different perspectives to ensure the walking balance of legged robots. Among different kinds of the currently developed control methods, the feedforward force control method, and the feedback control method based on the typical virtual model are most commonly seen.

Since the contact foot force is one of the critical factors that affect the attitude fluctuations, many scholars try to improve the walking balance of legged robots by planning desirable feedforward foot forces to ensure system force equilibrium. Song and Waldron [12] computed the feedforward foot forces of the ASV hexapod robot to improve its walking performance. In their method, constraints based on the system force equilibrium and internal force counteraction were established to get a unique solution of the foot force vector. Li et al. [13] proposed a dynamic force distribution approach for a quadruped robot with flexible joints. Through the force distribution approach, the desired feedforward foot forces to suppress the uncertainties of the dynamics model and perturbing forces were computed. Jiang et al. [14] proposed a foot force distribution method based on a pseudo-inverse formulation to reduce the risk of the foot slip. Galvez et al. [15] computed the desired feedforward foot forces of their quadruped robot SILO4 through the analysis of the static force equilibrium. Moosavian et al. [16] obtained the desired foot forces to achieve stable hexapod locomotion through an optimization way. In their method, the energy consumption was set as the optimization goal. During computation, constraints to reduce foot slip and ensure system force equilibrium were also employed. Wang et al. [17] employed a similar idea of energy consumption optimization. In their method, equality constraints of dynamic system force and moment equilibrium, and inequality constraints of joint torque limitations were introduced. By solving a standard quadratic programming (QP) problem, the unique ideal foot forces were obtained. Similar optimization ways to compute feedforward foot forces were also proposed by Roy et al. [18] and Mahapatra et al. [19] in developing their robots.

Undoubtedly, the feedforward foot force controllers can improve the walking balance of legged robots. Nevertheless, some obvious disadvantages can also be found. For a legged robot with more than three legs supporting the robot body simultaneously, a unique solution of the ideal foot force set is hard to obtain. To solve this redundancy problem, additional constraints and optimization methods are needed to be employed [20,21,22,23,24]. The computation is usually complex and nonlinear. In addition, although attitude fluctuations can be reduced through the regulation of the foot forces, the demand for walking attitude tracking cannot be satisfied only through a feedforward way. To further improve the walking balance of legged robots, control methods based on the typical virtual model are pushed forward by many scholars.

In the research field of virtual model controllers, a virtual spring–damper model is assumed to be assembled on the robot body of a legged robot. While walking, virtual forces generated by the virtual model are always acting on the robot body to regulate attitude deviations. Yoneda et al. [25] employed a virtual spring–damper model called sky–hook suspension to regulate the walking attitude of the robot TITAN VI. In their method, the virtual forces acting on the robot body were computed according to the feedback attitude data measured by gyroscopes. Huang et al. [26] proposed a method using sliding mode control to realize the active suspension effect based on the virtual spring–damper model. A similar control idea based on the virtual spring–damper model can be found in another work of Huang’s [27]. Wang et al. [28] employed the virtual model to regulate the attitude of a wheeled foot quadruped robot. In their control method, desired foot forces mapping from the virtual forces were calculated. Then, with the help of a position-based impedance model, the body attitude deviations were regulated through the regulation of the foot forces. Shi et al. [29] proposed a control scheme based on the virtual spring–damper model and impedance model. In their control scheme, the virtual forces acting on the robot body were generated by the virtual model and the desired motion state parameters. With the user-defined constraints and the impedance model, the desired joint torques were computed to regulate the walking attitude of their quadruped robot.

Compared with the feedforward foot force controllers, the virtual model controllers may achieve better control performances in attitude regulations of legged robots. Unlike the indirect attitude regulation way of the feedforward foot force controllers, the virtual forces are directly generated according to the attitude deviations under the function of the virtual model. Therefore, the demand for tracking user-defined attitude values can be better. Nevertheless, although the control performance of a virtual model controller may be good, the virtual model is still additionally added to the control system. The stiffness and damping coefficients on different degrees of freedom need to be well designed if a good walking performance of a legged robot wants to be ensured. The coefficient setting process is often inconvenient.

The motion of a legged robot is realized through the continuous switches between the support and swing statuses of the legs. For most currently developed legged robots, the support leg trajectory is usually designed only according to the motion direction and the desired walking velocity of the center of mass (CoM). During this process, the trajectories of the attitude angles are not taken into consideration [11,17,30,31]. This is why the motions of the support legs cannot adapt to the changes of the external terrain conditions, and additional balance control methods like the feedforward foot force control methods or the virtual model control methods are needed to be introduced into the control systems. In addition, in most currently developed balance walking controllers, the influence of the swing leg motions on the body attitude adjustment process is not considered. In fact, for many currently developed legged robots, the trajectory of the swing leg is only designed with different shapes according to the desired motion state of the robot in the robot body coordinate without considering the terrain attitude angles, namely the yaw, pitch, and roll angles of the terrain [12,32,33,34]. For a legged robot walking on flat terrain, this swing leg trajectory design is easy to be carried out and won’t cause any trouble to the walking performance. However, when facing the slope walking situation, this design may cause undesirable problems to the walking balance of a legged robot. For a legged robot walking on a slope, the landing foot position of a swing leg trajectory designed in the robot body coordinate without considering the terrain attitude angles cannot be changed according to the slope angle. If a special user-defined attitude angle of a legged robot is wanted to be ensured on a slope, the attitude adjustment process may lead to the early landings of the swing legs. Due to the unchangeable landing foot positions, the swing legs will keep moving to the planned landing positions. Then, the robot body will be lift up and forced to be parallel to the slope surface. Therefore, the attitude adjustment process will be hindered. The contradiction between the unchangeable landing foot positions of the swing legs and the attitude adjustment process will further affect the motion balance of the robot.

From the discussions of the currently developed balance walking controllers for legged robots, it can be found that if the attitude trajectories of a legged robot during walking are well designed to counteract the walking fluctuations, and the leg trajectories are generated with both the robot attitude and terrain attitude angles considered, both the walking balance and the simplicity of the control system of a legged robot can be ensured. To achieve this goal, a simple attitude trajectory optimization method to ensure balance motion control of a large-size hexapod robot is proposed in this paper. In this method, the optimization goal, namely the attitude trajectories to ensure balance walking of the large-size hexapod robot, are well planned first. During planning the attitude trajectories, both the desired walking attitude angles and the actual walking attitude angles of the robot are significantly considered to counteract the walking attitude fluctuations. Then, to ensure the optimized walking attitude trajectories can be realized through the motions of the legs, the leg trajectory generation method is developed. The support leg trajectory is automatically generated based on the optimized attitude trajectories to realize the walking attitude adjustment process. The terrain attitude angles which will affect the landing positions of the swing legs is significantly considered when designing the swing leg trajectory to reduce the bad influence of the swing legs on the robot body attitude adjustment process. Through the collaborative motions of the swing and support legs, the optimized walking attitude trajectories of the robot can be finally realized, and the balance walking of the robot can be ensured.

Unlike the currently developed balance controllers for legged robots, no additional control units like the feedforward foot force control unit or the virtual model unit are needed to be introduced into the control system. The control system can be simplified. In addition, through the optimized design of the swing leg trajectory with the terrain attitude angles considered, the robot can realize arbitrary attitude adjustment without being affected by the terrain changes.

The whole paper is organized as follows: in Section 2, the whole system of the large-size hexapod robot is overviewed, and the kinematics modeling process is introduced. In Section 3, the attitude trajectory optimization method to ensure balance hexapod locomotion is introduced in detail. In Section 4, comparative experiments carried out on the real large-size hexapod robot are introduced, and the experiment results to demonstrate the effectiveness of the attitude trajectory optimization method in ensuring balance hexapod locomotion are discussed.

## 2. System Overview and Kinematics Modeling of the Large-Size Hexapod Robot

### 2.1. The Brief Introduction of the Large-Size Hexapod Robot System

**Figure 1:**
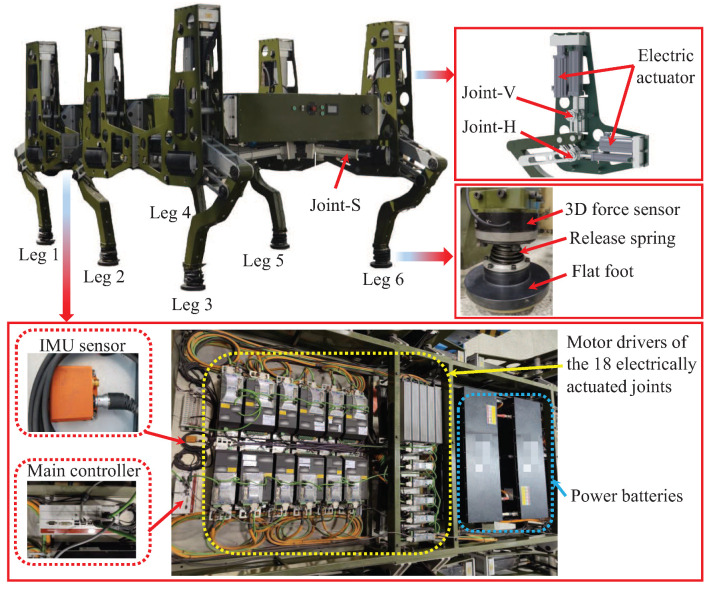
The overall structure of the large-size hexapod robot with eighteen electrically actuated joints.

To meet the demand of large-load bearing transportation in challenging outdoor environments, a large-size hexapod robot is developed, just as depicted in Figure 1. The size of the robot is designed to be over 4 m × 2.5 m × 2 m. The whole weight of the robot is 2.5 t. Six legs are symmetrically assembled on the body of the robot. The legs on the left side of the body are marked as leg 1, leg 2, and leg 3 from front to rear, while the right three legs are marked as leg 4, leg 5, and leg 6.

To facilitate the realization of large-load bearing and improve the control accuracy of the leg movement, a pantograph mechanism is employed while designing the leg structure. Three electrically driven joints are assembled on the robot leg, as the horizontal joint (Joint-H), the vertical joint (Joint-V), and the swing joint (Joint-S) shown in Figure 1. Each electric actuator of the joint is composed of a servo motor, a transmission mechanism, and an electric cylinder. Due to the motion-decoupling feature at the principal plane of the pantograph mechanism, the control of the robot leg is simple. For instance, the horizontal motion of the robot leg is only actuated by Joint-H with no motion disturbances from the other two joints.

The foot of the robot is designed to be flat to increase the contact area on the terrain during walking. The leg and the foot are connected by a spherical hinge to realize the flexible motion of the foot and improve the compliance of the foot with the terrain. A release spring is assembled outside the spherical hinge to ensure that, when the foot does not contact the terrain, it can automatically return to the original posture. A three-dimensional foot-force sensor is assembled on each foot of the robot to monitor the leg movement status and measure the contact foot forces.

Eighteen motor drivers of the joint servo motors, and the power battery packs are assembled on the robot body to provide the driving power. An inertial measurement unit (IMU) is assembled on the body of the robot to monitor the motion state of the robot (e.g., the body attitude changes and accelerations along different directions). The main motion controller of the robot is an industrial PC from Beckhoff (Beckhoff China, Shanghai, China) with an i5 CPU and a real-time system equipped. The control algorithms are written in the combination of the C++ language and the PLC language on the TwinCAT 3 plateform from Beckhoff. Fast data transmission can be ensured by using the Ethernet bus. Using remote desktop technology and WiFi connection, the robot operator can control the motion of the robot remotely and wirelessly.

### 2.2. Kinematics Modeling of the Large-Size Hexapod Robot

Just as discussed above, the structure of the robot leg is designed as a pantograph mechanism. Due to the advantage of the motion-decoupling feature of this mechanism, the forward and inverse kinematics models of the robot can be easily established only through geometric analysis.

**Figure 2:**
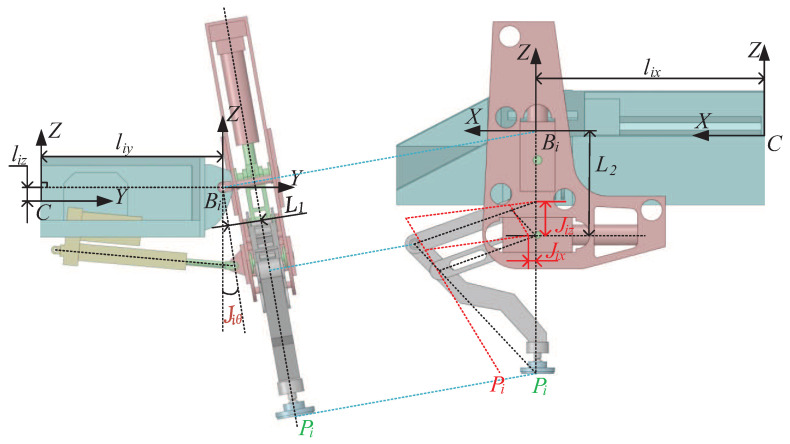
The schematic diagram of the robot leg structure and coordinate frame definitions of the hexapod robot.

As shown in Figure 2, two kinds of coordinates are defined to establish the kinematics models of the robot, which are the base coordinate $B_{i}$ of leg *i* located in the hinged point of the swing joint, and the robot body coordinate *C* situated in the geometric center of the robot body. Through the geometric analysis, the forward and inverse kinematics models of the robot can be established, as shown in Equations (1) and (2), respectively:

(1) $$\left\{\begin{array}{c} {}^{C}P_{ix}=K_{x}J_{ix}+l_{ix}\\ {}^{C}P_{iy}=L_{1}\cos\left(J_{i\theta }\right)+\left(K_{z}J_{iz}+L_{2}\right)\sin\left(J_{i\theta }\right)+l_{iy}\\ {}^{C}P_{iz}=L_{1}\sin\left(J_{i\theta }\right)-\left(K_{z}J_{iz}+L_{2}\right)\cos\left(J_{i\theta }\right)+l_{iz} \end{array}\right.$$

(2) $$\left\{\begin{array}{c} J_{ix}=\frac{{}^{B_{i}}P_{ix}}{K_{x}}\\ J_{iz}=\frac{\sqrt{{}^{B_{i}}{P_{iy}}^{2}+{}^{B_{i}}{P_{iz}}^{2}-{L_{1}}^{2}}-L_{2}}{K_{z}}\\ J_{i\theta }=\arcsin\left(\frac{{}^{B_{i}}P_{iz}L_{1}+{}^{B_{i}}P_{iy}\sqrt{{}^{B_{i}}{P_{iy}}^{2}+{}^{B_{i}}{P_{iz}}^{2}-{L_{1}}^{2}}}{{}^{B_{i}}{P_{iy}}^{2}+{}^{B_{i}}{P_{iz}}^{2}}\right) \end{array}\right.$$

with

(3) $${}^{B_{i}}P_{i}={}^{C}P_{i}-l_{i}={\left[{}^{C}P_{ix}-l_{ix},{}^{C}P_{iy}-l_{iy},{}^{C}P_{iz}-l_{iz}\right]}^{T}$$

where $J_{i}={\left[J_{ix},J_{iz},J_{i\theta }\right]}^{T}$ represents the equivalent joint position vector of leg *i*. ${}^{B_{i}}P_{i}={\left[{}^{B_{i}}P_{ix},{}^{B_{i}}P_{iy},{}^{B_{i}}P_{iz}\right]}^{T}$ and ${}^{C}P_{i}={\left[{}^{C}P_{ix},{}^{C}P_{iy},{}^{C}P_{iz}\right]}^{T}$ represent the foot position vectors of leg *i* in coordinate $B_{i}$ and coordinate *C*, respectively. $l_{i}={\left[l_{ix},l_{iy},l_{iz}\right]}^{T}$ represents the position vector of $B_{i}$ in the body coordinate *C*. $K_{x}$ and $K_{z}$ represent the extension coefficients of the horizontal and vertical joints, respectively. $L_{1}$ represents the distance from the origin of $B_{i}$ to the parallelogram plane. $L_{2}$ represents the distance from the projection of the origin of $B_{i}$ in the parallelogram plane to the central axis of the horizontal joint.

Due to the simple forms of the kinematics models of the robot, the relationship between the foot position of one leg and the related joints can be precisely determined. Therefore, leg motion can be easily controlled through a traditional PID way at the joint level.

## 3. Attitude Trajectory Optimization Method to Ensure Balance Hexapod Locomotion

**Figure 3:**
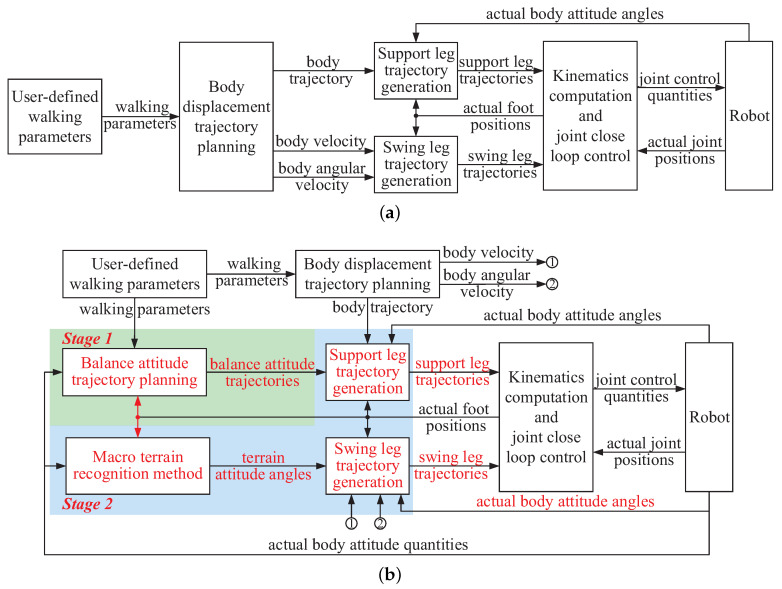
The basic control scheme of a legged robot, and the control scheme of the attitude trajectory optimization method proposed for the large-size hexapod robot: (**a**) the basic control scheme of a legged robot and (**b**) the control scheme of the attitude trajectory optimization method proposed.

Usually, during a basic walking process of a legged robot, the body displacement trajectory is planned based on the user-defined walking parameters. Based on this trajectory, the trajectories of the support legs are generated. The trajectories of the swing legs are designed in the body coordinate with only the body velocity and angular velocity considered. Then, through the kinematics computation and joint close loop control, the robot is driven to move with no walking attitude regulation. The basic control scheme which is commonly employed for a legged robot is shown in Figure 3a. Due to the none regulation of the walking attitude, the walking balance of the robot cannot be ensured.

To ensure a balance hexapod locomotion of the large-size hexapod robot, the body attitude angles during walking must be optimized and controlled within the desired ranges, especially when the external terrain is uneven. To achieve this goal, in this section, an attitude trajectory optimization method is introduced. The basic control scheme of this method is shown in Figure 3b. The attitude trajectory optimization process mainly consists of two stages. The first stage is the balance attitude trajectory planning, and the second stage is the leg trajectory generation. During stage 1, the optimization goal, namely the body attitude trajectories which are not considered in the commonly used control scheme of a legged robot is well planned first. Both the actual body attitude quantities (the body attitude angles, the body attitude angular velocities and the body attitude angular accelerations obtained by the IMU feedback signals), and the desired body attitude quantities are employed when planning the balance attitude trajectories to reduce the walking attitude fluctuations. During stage 2, the trajectories of the support and swing legs are generated. The support leg trajectory is automatically generated based on the planned balance attitude trajectories and the body displacement trajectory to regulate the walking process and satisfy the demand of the planned attitude trajectories. When designing the swing leg trajectory, the terrain attitude angles which are not considered during a common swing leg trajectory designing process are significantly considered to reduce the bad influence of the swing legs on the robot body attitude adjustment process (the method to obtain the terrain attitude angles is introduced in Section 3.2.1). Based on the collaborative motions of the swing and support legs, the walking attitude trajectories are continually optimized during walking, and the walking balance of the large-size hexapod robot can be ensured.

### 3.1. Stage 1: Balance Attitude Trajectory Planning

#### 3.1.1. Balance Attitude Trajectory Planning via High Order Polynomial Interpolation

Usually, the parameters to evaluate the balance motion performance of the robot are represented by the attitude angles, namely the body Euler angles in Cartesian space. Good attitude angle trajectory planning will increase the motion performance of the hexapod robot. To achieve this goal, an attitude trajectory planning method via high order polynomial interpolation is developed. A general symbol τ is used to represent the attitude angles, where τ can be the yaw angle α, the pitch angle β, and the roll angle γ. The trajectories of α, β, and γ are planned through the same method introduced below.

**Figure 4:**
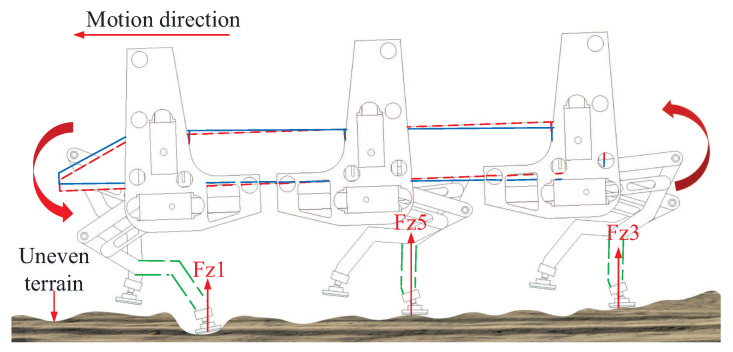
The robot body attitude change under unpredictable motion conditions.

During a hexapod walking process, especially when the large-size robot is walking on soft terrain, τ will be very easily changed by some unpredictable disturbances (e.g., the deformations of the robot legs, the deformation of the terrain and the big contact impacts between the robot legs and the terrain), as shown in Figure 4. If small body attitude deviations are not adjusted in time, the deviations will increase as the robot moves further. Even worse, the robot may lose motion stability, and irreversible damage may occur. To solve this problem, a 6-order polynomial interpolation method is employed to plan the balance angle attitude trajectory of the robot. The planned attitude trajectory is shown in Equation (4):

(4) $$\tau_{t_{r}}=a_{0}+a_{1}t_{r}+a_{2}{t_{r}}^{2}+a_{3}{t_{r}}^{3}+a_{4}{t_{r}}^{4}+a_{5}{t_{r}}^{5}+a_{6}{t_{r}}^{6}$$

where $\tau_{t_{r}}$ represents the planned attitude angle trajectory. $t_{r}$ represents the planning time, $t_{r}\in \left[0,T_{r}\right]$. $T_{r}$ represents the user-defined attitude deviation adjustment time. $a_{0}$, $a_{1}$, $a_{2}$, $a_{3}$, $a_{4}$, $a_{5}$, and $a_{6}$ represent the polynomial parameters which need to be determined.

Based on Equation (4), the attitude angular velocity trajectory and angular acceleration trajectory can be obtained using the differential method, as shown in Equations (5) and (6), respectively:

(5) $$v\tau_{t_{r}}=a_{1}+2a_{2}t_{r}+3a_{3}{t_{r}}^{2}+4a_{4}{t_{r}}^{3}+5a_{5}{t_{r}}^{4}+6a_{6}{t_{r}}^{5}$$

(6) $$a\tau_{t_{r}}=2a_{2}+6a_{3}t_{r}+12a_{4}{t_{r}}^{2}+20a_{5}{t_{r}}^{3}+30a_{6}{t_{r}}^{4}$$

To get a smooth and continuous attitude angle trajectory, certain constraints must be satisfied. Usually, at the beginning of an attitude deviation adjustment cycle, namely at the time $t_{r}=0$, the initial attitude angle, angular velocity, and angular acceleration values can be obtained according to the IMU sensor feedback signals. At the end of a deviation adjustment cycle, namely when $t_{r}=T_{r}$, the desired attitude angle value is user-defined and wanted to be tracked. At the same time, the desired angular velocity, and angular acceleration are usually wanted to be zero to make the angle trajectory smooth and reduce the attitude deviation accumulation. Therefore, at the time $t_{r}=0$ and $t_{r}=T_{r}$, six constraints for planning the attitude angle trajectory can be set up. To further make the planned attitude trajectory smooth, the middle position of the attitude angle trajectory during a deviation adjustment cycle is also constrained. Therefore, seven constraints that the planned attitude angle trajectory must satisfy can be set up, as shown in Equation (7):

(7) $$\left[\begin{array}{cc} \tau_{0}=\tau_{ini} & \tau_{T_{r}}=\tau_{dir}\\ v\tau_{0}=v\tau_{ini} & v\tau_{T_{r}}=0\\ a\tau_{0}=a\tau_{ini} & a\tau_{T_{r}}=0\\ \tau_{mid}=\left(\tau_{ini}+\tau_{dir}\right)/2 & \end{array}\right]$$

where $\tau_{ini}$, $v\tau_{ini}$, and $a\tau_{ini}$ represent the initial attitude angle, angular velocity and angular acceleration values obtained according to the IMU sensor feedback signals, respectively. $\tau_{dir}$ represents the desired attitude angle which is user-defined. $\tau_{mid}$ represents the middle attitude angle at the time $t_{r}=T_{r}/2$.

Then, according to Equations (4)–(7), the polynomial parameters in Equation (4) can be finally determined, as shown in Equation (8).

(8) $$\left[\begin{array}{c} a_{0}\\ a_{1}\\ a_{2}\\ a_{3}\\ a_{4}\\ \begin{array}{c} a_{5}\\ a_{6} \end{array} \end{array}\right]={\left[\begin{array}{ccccccc} 1 & 0 & 0 & 0 & 0 & 0 & 0\\ 1 & T_{m} & {T_{m}}^{2} & {T_{m}}^{3} & {T_{m}}^{4} & {T_{m}}^{5} & {T_{m}}^{6}\\ 1 & T_{r} & {T_{r}}^{2} & {T_{r}}^{3} & {T_{r}}^{4} & {T_{r}}^{5} & {T_{r}}^{6}\\ 0 & 1 & 0 & 0 & 0 & 0 & 0\\ 0 & 1 & 2T_{r} & 3{T_{r}}^{2} & 4{T_{r}}^{3} & 5{T_{r}}^{4} & 6{T_{r}}^{5}\\ 0 & 0 & 2 & 0 & 0 & 0 & 0\\ 0 & 0 & 2 & 6T_{r} & 12{T_{r}}^{2} & 20{T_{r}}^{3} & 30{T_{r}}^{4} \end{array}\right]}^{-1}\left[\begin{array}{c} \tau_{ini}\\ \begin{array}{c} \tau_{mid}\\ \tau_{dir} \end{array}\\ v\tau_{ini}\\ 0\\ a\tau_{ini}\\ 0 \end{array}\right]$$

where $T_{m}$ = $T_{r}$/2.

Based on the polynomial parameters computed from Equation (8), the planned attitude angle trajectory can be obtained through the computation of Equation (4).

#### 3.1.2. Constraints of the Desired Attitude Angle Values and the Deviation Adjustment Time

According to the attitude trajectory planning process, only the desired attitude angle $\tau_{dir}$ and the deviation adjustment time $T_{r}$ need to be set by the robot users. When setting $\tau_{dir}$ and $T_{r}$, the leg driving ability must be taken into consideration. The adjustable attitude angle domains are strictly limited by the motion ranges of the joints, while the minimum $T_{r}$ is strictly limited by the maximum joint moving velocities.

During a full attitude adjustment cycle, the relationship between the foot position of leg *i* and the adjustable attitude angles can be set up, as shown in Equation (9):

(9) $${}^{C}{P_{i}}^{\prime }=R_{W}{}^{C}P_{i0}+\Delta Q$$

with

(10) $$\left\{\begin{array}{c} R_{W}=\left[\begin{array}{ccc} b_{1}b_{3} & b_{1}b_{4}b_{6}-b_{2}b_{5} & b_{1}b_{4}b_{5}+b_{2}b_{6}\\ b_{2}b_{3} & b_{2}b_{4}b_{6}+b_{1}b_{5} & b_{2}b_{4}b_{5}-b_{1}b_{6}\\ -b_{4} & b_{3}b_{6} & b_{3}b_{5} \end{array}\right]\\ b_{1}=\cos\left(\Delta \alpha \right)\\ b_{2}=\sin\left(\Delta \alpha \right)\\ b_{3}=\cos\left(\Delta \beta \right)\\ b_{4}=\sin\left(\Delta \beta \right)\\ b_{5}=\cos\left(\Delta \gamma \right)\\ b_{6}=\sin\left(\Delta \gamma \right) \end{array}\right.$$

where $R_{w}$ represents the rotation transformation matrix of the adjusted body coordinate at time $t_{r}$ = $T_{r}$ with respect to the original body coordinate at time $t_{r}=0$. ${}^{C}P_{i0}={\left[{}^{C}P_{ix0},{}^{C}P_{iy0},{}^{C}P_{iz0}\right]}^{T}$ and ${}^{C}{P_{i}}^{\prime }={\left[{}^{C}P_{ix}^{\prime },{}^{C}P_{iy}^{\prime },{}^{C}P_{iz}^{\prime }\right]}^{T}$ represent the foot positions at time $t_{r}=0$ and $t_{r}$ = $T_{r}$ in the body coordinate *C*, respectively. Δα, Δβ and Δγ represent the adjustable body yaw, pitch and roll angles, respectively. $\Delta Q={\left[\Delta Q_{x},\Delta Q_{y},\Delta Q_{z}\right]}^{T}$ represents the robot body displacement vector during one attitude adjustment cycle.

Because of the very short deviation adjustment time, ΔQ can be expressed as shown in Equation (11):

(11) $$\Delta Q={}^{W}V\;\cdot \;T_{r}$$

where ${}^{W}V={\left[{}^{W}V_{x},{}^{W}V_{y},{}^{W}V_{z}\right]}^{T}$ represents the global moving velocity vector of the robot.

Using Equation (1), ${}^{C}P_{i0}$ in Equation (9) can be easily computed based on the feedback joint position signals. However, ${}^{C}{P_{i}}^{\prime }$ in Equation (9) is strictly limited by the motion ranges of the joints. During one attitude adjustment cycle, the joint motions should be kept within their motion ranges, as shown in Equation (12):

(12) $$J_{i}\in \left[J_{i\min},J_{i\max}\right]$$

where $J_{i\min}$ and $J_{i\max}$ represent the joint limit positions of leg *i*.

Then, based on Equations (1) and (12), the domains of ${}^{C}{P_{i}}^{\prime }$ can be easily computed, as shown in Equation (13):

(13) $${}^{C}{P_{i}}^{\prime }\in E_{i}^{p}$$

where $E_{i}^{p}$ represents the domains of ${}^{C}{P_{i}}^{\prime }$.

To get the domains of the adjustable attitude angles in one adjustment cycle, the current support state of the hexapod robot should be taken into consideration. Depending on the number of the support legs, the support states of the hexapod robot can be divided into many types. If the number of support legs is three, and the support legs are assumed to be leg 1, leg 3, and leg 5, the transformation matrix $R_{w}$ in Equation (9) can be expressed into Equation (14):

(14) $${\left\{\begin{array}{c} R_{W}=\left[\begin{array}{ccc} {P_{1}}^{\prime } & {P_{3}}^{\prime } & {P_{5}}^{\prime } \end{array}\right]\left[\begin{array}{ccc} {}^{C}P_{10} & {}^{C}P_{30} & {}^{C}P_{50} \end{array}\right]\\ {P_{1}}^{\prime }={}^{C}{P_{1}}^{\prime }-\Delta Q\\ {P_{3}}^{\prime }={}^{C}{P_{3}}^{\prime }-\Delta Q\\ {P_{5}}^{\prime }={}^{C}{P_{5}}^{\prime }-\Delta Q \end{array}\right.}^{-1}$$

Based on Equations (10), (11), (13), and (14), the domains of the adjustable attitude angles can be computed, as shown in Equation (15). For other three-legged support states, the computation process of the adjustable attitude angle domains is the same as the support state with leg 1, leg 3, and leg 5 supporting the robot:

(15) $$\left\{\begin{array}{c} \Delta \alpha \in \left[\Delta \alpha_{\min},\Delta \alpha_{\max}\right]\\ \Delta \beta \in \left[\Delta \beta_{\min},\Delta \beta_{\max}\right]\\ \Delta \gamma \in \left[\Delta \gamma_{\min},\Delta \gamma_{\max}\right] \end{array}\right.$$

As α, β, or γ is represented by τ, Equation (15) is represented by Equation (16):

(16) $$\Delta \tau \in E^{\tau }=\left[\Delta \tau_{\min},\Delta \tau_{\max}\right]$$

where $E^{\tau }$ represents the domain of the adjustable attitude angle. $\Delta \tau_{min}$ and $\Delta \tau_{max}$ represent the boundary values of the adjustable attitude angle domain under the joint motion range constraint of the current support state, respectively.

**Figure 5:**
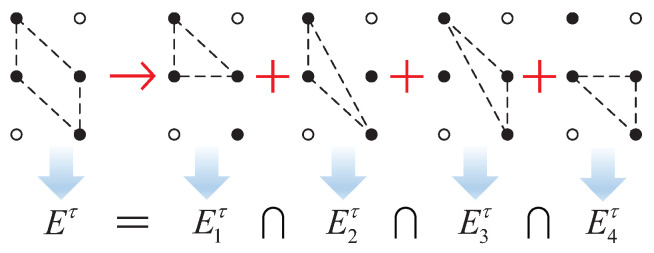
The divided three-legged support states of the four-legged support state.

For the support states with more than three support legs, the domain of the adjustable attitude angle can be computed through a similar way. Figure 5 shows a typical support state with four support legs, where the solid black dot represents the support leg while the white hollow dot represents the swing leg. It can be seen from Figure 5 that the original support state with more than three support legs can be divided into *N* kinds of three-legged support states. For each three-legged support state, the domain of the adjustable attitude angle, namely $E_{n}^{\tau }$, can be computed through the way discussed above. Then, the final domain of the adjustable attitude angle can be computed by calculating the intersection set of $E_{n}^{\tau }$, and the adjustable attitude angle should satisfy this domain, just as shown in Equation (17):

(17) $$\left\{\begin{array}{c} \Delta \tau \in \overset{N}{\underset{n=1}{\cap }}E_{n}^{\tau }=\left[\Delta \tau_{\min},\Delta \tau_{\max}\right]\\ E_{n}^{\tau }=\left[\Delta \tau_{n\min},\Delta \tau_{n\max}\right]\\ \Delta \tau_{\min}=\max\left(\Delta \tau_{n\min}\right)\\ \Delta \tau_{\max}=\min\left(\Delta \tau_{n\max}\right) \end{array}\right.$$

where *n* represents the label number of each three-legged support state. *N* represents the total number of three-legged support states. $\Delta \tau_{\min}$ and $\Delta \tau_{\max}$ represent the boundary values of the final domain of the adjustable attitude angle. $E_{n}^{\tau }$ represents the adjustable attitude angle domain of each three-legged support state. $\Delta \tau_{n\min}$, and $\Delta \tau_{n\max}$ represent the boundary values of $E_{n}^{\tau }$.

Based on Equation (17), the desired attitude angle domain can be obtained, and the desired attitude angle must be set within the domain, just as shown in Equation (18):

(18) $$\tau_{dir}\in E=\left[\tau_{ini}+\Delta \tau_{\min},\tau_{ini}+\Delta \tau_{\max}\right]$$

where $E=\left[\tau_{ini}+\Delta \tau_{\min},\tau_{ini}+\Delta \tau_{\max}\right]$ represents the domain of the desired attitude angle.

During every attitude adjustment cycle, the robot examines the three desired attitude angles set by the robot users to judge if they are within their domains. If one desired attitude angle is set out of its domain, the robot chooses the boundary value to be the new desired attitude angle to continue the attitude adjustment process. The selection of the maximum or minimum boundary value depends on the direction of the attitude adjustment process.

Another important user-defined parameter which will affect the attitude adjustment performance is the deviation adjustment time $T_{r}$. If $T_{r}$ is set too long, the balance hexapod locomotion will struggle to be ensured. Conversely, the joint actuators may reach the maximum driving velocities and cause bad situations like the robot shaking. According to the attitude trajectory planning process, the attitude angular velocity trajectories may not be straight lines. In other words, the adjustable angular velocities may not be uniform. Therefore, it is difficult to get a definite mapping between the joint driving velocity and $T_{r}$. Thus, $T_{r}$ should be set by the robot users according to the actual locomotion performance, just like the tuning process of PID parameter adjustment in conventional PID control systems. During the tuning process of $T_{r}$, $T_{r}$ should also be kept within a domain, as shown in Equation (19):

(19) $$\left\{\begin{array}{c} T_{r}\in \left[\Delta t,T_{\sup}\right]\\ T_{\sup}=\lambda \;\cdot \;T \end{array}\right.$$

where Δt represents the sample time of the hexapod robot. $T_{\sup}$ represents the support time during one gait cycle. λ represents the duty factor of the support legs. *T* represents the gait cycle time.

### 3.2. Stage 2: Leg Trajectory Generation

#### 3.2.1. Swing Leg Trajectory Generation

**Figure 6:**
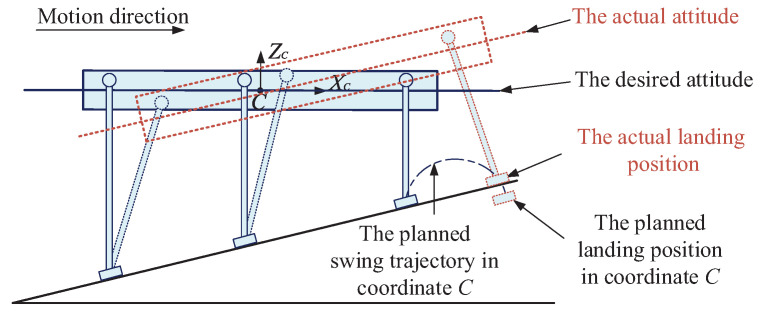
The diagram of the attitude adjustment obstruction caused by the traditional swing trajectory design.

As discussed in the introduction, for most currently developed legged robots, the swing leg trajectory is usually designed in the body coordinate *C* without considering the terrain attitude angles. When facing the slope walking situation, the early landings of the swing legs may occur. The swing legs with the unchangeable landing positions with respect to the robot body coordinate *C* will keep moving to the planned landing positions and force the robot body to be parallel to the slope surface. Therefore, the attitude adjustment process with special user-defined desired attitude angles will be hindered. The contradiction between the unchangeable landing positions of the swing legs and the attitude adjustment process will further affect the motion balance of the robot, as shown in Figure 6.

**Figure 7:**
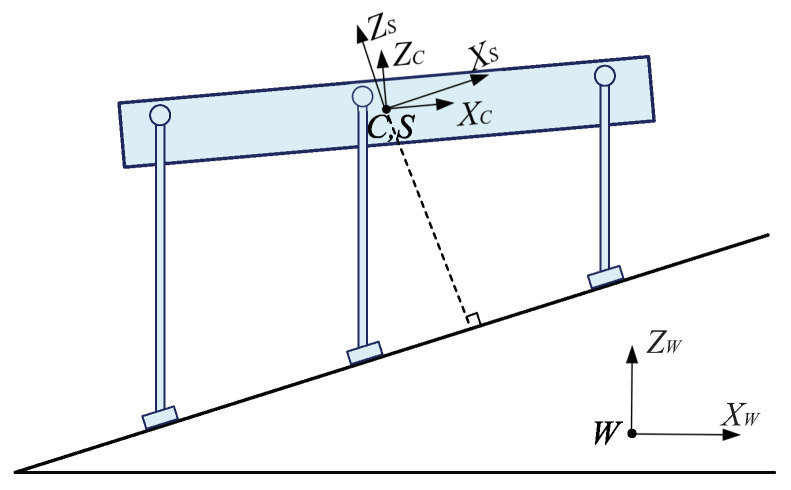
The diagram of the coordinates defined.

To solve this problem, a swing leg trajectory generation method with terrain attitude angles considered is proposed. For a slope-climbing legged robot, the attitude angles of the slope are important factors which will influence the walking balance of the robot. Therefore, to get a swing leg trajectory which can adapt to the terrain, the slope attitude angles are needed to be obtained first. Similar to the coordinates defined in the authors’ previous work [35] (see Figure 3 in reference [35]), a slope coordinate *S* and the global coordinate *W* are defined to obtain the slope attitude angles, as shown in Figure 7. The origin of the slope coordinate *S* is located at the origin of the body coordinate *C*. The *z*-axis of coordinate *S*, namely the $Z_{S}$-axis shown in Figure 7, is defined to be perpendicular to the slope surface. The *x*-axis of coordinate *S*, namely the $X_{S}$-axis shown in Figure 7, is defined to be parallel to the slope surface. The yaw angle of the slope is defined as the same as the yaw angle of the robot. The *z*-axis of the global coordinate *W*, namely the $Z_{W}$-axis shown in Figure 7, is defined to be perpendicular to the horizontal plane. The *x*-axis of coordinate *W*, namely the $X_{W}$-axis shown in Figure 7, is defined to be parallel to the horizontal plane with the same direction as the initial *x*-direction of coordinate *C*. The *y*-axis of coordinate *W* is defined to be perpendicular to axis $Z_{W}$ and axis $X_{W}$, with the same direction as the initial *y*-direction of coordinate *C*. The coordinate *W* is an absolute coordinate and can be located anywhere according to the robot users’ commands.

In the authors’ previous work [35], a macro terrain recognition method is proposed to obtain the slope plane based on the foot positions of the support legs. Through the macro terrain recognition method, the unit normal vector of the slope ${}^{W}k_{S}$ = ${\left[{}^{W}k_{Sx},{}^{W}k_{Sy},{}^{W}k_{Sz}\right]}^{T}$ can be calculated using Equation (4) shown in [35]. Employing the same ${}^{W}k_{S}$ in reference [35], the relationship between ${}^{W}k_{W}$ = ${\left[0,0,1\right]}^{T}$ which represents the unit normal vector of the global coordinate *W*, and ${}^{W}k_{S}$ can be obtained, as shown in Equation (20):

(20) $${}^{W}k_{S}={}_{S}^{W}R^{\prime }\;\cdot \;{}^{W}k_{W}$$

where ${}_{S}^{W}R^{\prime }$ represents the rotation transformation matrix of the slope coordinate *S* with respect to the global coordinate *W*. ${}_{S}^{W}R^{\prime }$ has the same form as $R_{w}$ shown in Equation (10), and consists of the actual yaw angle $\alpha_{S}^{\prime }$, the actual pitch angle $\beta_{S}^{\prime }$, and the actual roll angle $\gamma_{S}^{\prime }$ of the slope coordinate *S*.

During walking, the actual yaw angles of the body coordinate *C* and the slope coordinate *S* are the same. Based on this, by solving Equation (20), the attitude angles of the slope coordinate *S* can be obtained, as shown in Equation (21):

(21) $$\left\{\begin{array}{c} \alpha_{S}^{\prime }=\alpha^{\prime }\\ \beta_{S}^{\prime }=\arctan\left(\frac{{}^{W}k_{Sx}\;\cdot \;\cos\alpha^{\prime }+{}^{W}k_{Sy}\;\cdot \;\sin\alpha^{\prime }}{{}^{W}k_{Sz}}\right)\\ \gamma_{S}^{\prime }=\arcsin\left({}^{W}k_{Sx}\;\cdot \;\sin\alpha^{\prime }-{}^{W}k_{Sy}\;\cdot \;\cos\alpha^{\prime }\right) \end{array}\right.$$

where $\alpha^{\prime }$ represents the actual yaw angle of the robot during walking.

During a slope-climbing process, if a special body attitude of the hexapod robot is wanted to be ensured instead of being parallel to the slope surface at any time, the swing leg trajectory should be designed in the slope coordinate *S* to reduce the risk of lifting up the body. However, the swing leg trajectory must be finally generated through the inverse kinematics computations, and the inverse kinematics model of the robot is defined in the body coordinate *C*. Therefore, theoretically, the swing leg trajectory should be designed in the body coordinate *C*. To solve this conflict, assuming that the robot body is parallel to the slope. Based on this assumption, the swing leg trajectory designed in the slope coordinate *S* is the same as that in the body coordinate *C*. Then, combined with the actual attitude angles of the slope and the body, the swing leg trajectory can be transformed from the slope coordinate *S* to the actual body coordinate *C*.

To achieve a smooth trajectory of the swing leg, and reduce the velocity change brought by the leg status switching, a similar 6-order polynomial interpolation method to the attitude trajectory planning process is employed to design the swing leg trajectory in the slope coordinate *S*, as shown in Equation (22):

(22) $${}^{S}P_{ir}=a_{r0}+a_{r1}t+a_{r2}t^{2}+a_{r3}t^{3}+a_{r4}t^{4}+a_{r5}t^{5}+a_{r6}t^{6}$$

where $a_{r0}$, $a_{r1}$, $a_{r2}$, $a_{r3}$, $a_{r4}$, $a_{r5}$, and $a_{r6}$ represent the polynomial parameters which need to be determined. *r* represents the direction of the slope coordinate *S*, and *r* can be *x*, *y*, or *z*.

Based on Equation (22), the velocity trajectory and acceleration trajectory of the swing leg along the *r*-direction of the slope coordinate *S* can be obtained, as shown in Equations (23) and (24), respectively.

(23) $${}^{S}vP_{ir}=a_{r1}+2a_{r2}t+3a_{r3}t^{2}+4a_{r4}t^{3}+5a_{r5}t^{4}+6a_{r6}t^{5}$$

(24) $${}^{S}aP_{ir}=2a_{r2}+6a_{r3}t+12a_{r4}t^{2}+20a_{r5}t^{3}+30a_{r6}t^{4}$$

To obtain the unknown polynomial parameters and generate the swing leg trajectory, seven trajectory parameters of the swing leg should be designed, which are the starting position, velocity, and acceleration of the foot at the time $t_{1}=0$, the landing position, velocity, and acceleration of the foot at the time $t_{3}=\left(1-\lambda \right)T$, and the middle position of the foot at the time $t_{2}=\left(t_{1}+t_{3}\right)/2$. Based on the seven trajectory parameters and their corresponding times, the unknown polynomial parameters can be computed, as shown in Equation (25):

(25) $$\left[\begin{array}{c} a_{r0}\\ a_{r1}\\ a_{r2}\\ a_{r3}\\ a_{r4}\\ a_{r5}\\ a_{r6} \end{array}\right]={\left[\begin{array}{ccccccc} 1 & 0 & 0 & 0 & 0 & 0 & 0\\ 1 & t_{2} & {t_{2}}^{2} & {t_{2}}^{3} & {t_{2}}^{4} & {t_{2}}^{5} & {t_{2}}^{6}\\ 1 & t_{3} & {t_{3}}^{2} & {t_{3}}^{3} & {t_{3}}^{4} & {t_{3}}^{5} & {t_{3}}^{6}\\ 0 & 1 & 0 & 0 & 0 & 0 & 0\\ 0 & 1 & 2t_{3} & 3{t_{3}}^{2} & 4{t_{3}}^{3} & 5{t_{3}}^{4} & 6{t_{3}}^{5}\\ 0 & 0 & 2 & 0 & 0 & 0 & 0\\ 0 & 0 & 2 & 6t_{3} & 12{t_{3}}^{2} & 20{t_{3}}^{3} & 30{t_{3}}^{4} \end{array}\right]}^{-1}\left[\begin{array}{c} {}^{S}P_{ir1}\\ {}^{S}P_{ir2}\\ {}^{S}P_{ir3}\\ {}^{S}vP_{ir1}\\ {}^{S}vP_{ir3}\\ {}^{S}aP_{ir1}\\ {}^{S}aP_{ir3} \end{array}\right]$$

where ${}^{S}P_{ir1}$, ${}^{S}vP_{ir1}$ and ${}^{S}aP_{ir1}$ represent the starting foot position, velocity, and acceleration of the swing leg trajectory along the *r*-direction of the slope coordinate *S*, respectively. ${}^{S}P_{ir3}$, ${}^{S}vP_{ir3}$ and ${}^{S}aP_{ir3}$ represent the landing foot position, velocity, and acceleration of the swing leg trajectory along the *r*-direction of coordinate *S*, respectively. ${}^{S}P_{ir2}$ represents the middle foot position of the swing leg trajectory along the *r*-direction of coordinate *S*.

The starting foot position of a swing leg is the ending foot position of the same support leg. Therefore, based on the actual ending joint positions of the support leg *i*, the actual starting foot position of the swing leg *i* at the time $t_{1}=0$ in the body coordinate *C* can be obtained through the computation of Equation (1). Then, the starting foot position of the swing leg *i* in the slope coordinate *S* can be obtained, as shown in Equation (26):

(26) $${}^{S}P_{i1}={}_{S}^{W}{R^{\prime }}^{T}\;\cdot \;{}_{C}^{W}R^{\prime }\;\cdot \;{}^{C}P_{i1}$$

where ${}^{C}P_{i1}={\left[{}^{C}P_{ix1},{}^{C}P_{iy1},{}^{C}P_{iz1}\right]}^{T}$ represents the starting foot position vector of the swing leg *i* in the body coordinate *C* at the time $t_{1}=0$. ${}_{C}^{W}R^{\prime }$ represents the rotation transformation matrix of the body coordinate *C* with respect to the global coordinate *W*. ${}_{C}^{W}R^{\prime }$ has the same form as $R_{w}$ shown in Equation (10), and consists of the actual yaw, pitch, and roll angles of the robot during walking. ${}^{S}P_{i1}={\left[{}^{S}P_{ix1},{}^{S}P_{iy1},{}^{S}P_{iz1}\right]}^{T}$ represents the starting foot position vector of the swing leg *i* in the slope coordinate *S* at the time $t_{1}=0$.

Based on ${}^{S}P_{i1}$, ${}^{S}vP_{i1}={\left[{}^{S}vP_{ix1},{}^{S}vP_{iy1},{}^{S}vP_{iz1}\right]}^{T}$ which represents the starting foot velocity of the swing leg *i*, and ${}^{S}aP_{i1}={\left[{}^{S}aP_{ix1},{}^{S}aP_{iy1},{}^{S}aP_{iz1}\right]}^{T}$, which represents the starting foot acceleration of the swing leg *i* can be computed using the differential method, as shown in Equation (27):

(27) $$\left\{\begin{array}{c} {}^{S}vP_{i1}=\frac{d}{dt}{}^{S}P_{i1}\\ {}^{S}aP_{i1}=\frac{d}{dt}{}^{S}vP_{i1} \end{array}\right.$$

**Figure 8:**
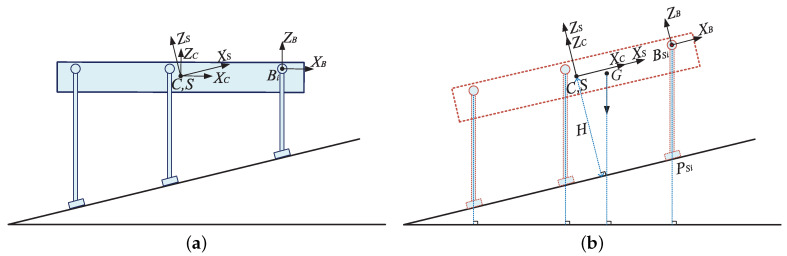
The hypothetical and actual postures of the robot standing still: (**a**) the actual robot posture and (**b**) the hypothetical robot posture.

To obtain the landing foot position, velocity, and acceleration of the swing leg, a hypothetical foot position of a support leg relative to the robot body when the robot is standing still is designed first. Assuming that the robot body is parallel to the slope when the robot is standing still on the slope, a hypothetical base coordinate $B_{Si}$ of leg *i* with the same directions as the slope coordinate can be obtained, as shown in Figure 8b. At this moment, when the line between the foot and the hypothetical base coordinate of the leg is parallel to the direction of the gravity, the projection of the CoM of the robot body in the polygon formed by the support feet is close to the center of the support polygon, and better stability of the robot can be ensured. Based on this hypothetical posture of the robot, the foot position of leg *i* in coordinate $B_{Si}$ can be obtained, as shown in Equation (28):

(28) $$\left[\begin{array}{c} 0\\ 0\\ k \end{array}\right]={}_{S}^{W}R^{\prime }\;\cdot \;\left[\begin{array}{c} {}^{B_{Si}}P_{Six}\\ {}^{B_{Si}}P_{Siy}\\ {}^{B_{Si}}P_{Siz} \end{array}\right]$$

where *k* represents the equivalent leg length. ${}^{B_{Si}}P_{Si}={\left[{}^{B_{Si}}P_{Six},{}^{B_{Si}}P_{Siy},{}^{B_{Si}}P_{Siz}\right]}^{T}$ represents the foot position vector of leg *i* in coordinate $B_{Si}$.

The robot body height *H* is usually set by the robot users; therefore, ${}^{B_{Si}}P_{Siz}$ can be calculated as shown in Equation (29):

(29) $${}^{B_{Si}}P_{Siz}=-\left(H+l_{iz}\right)$$

By substituting Equation (29) into Equation (28), ${}^{B_{Si}}P_{Six}$ and ${}^{B_{Si}}P_{Siy}$ can be calculated, as shown in Equation (30):

(30) $$\left\{\begin{array}{c} k=-\left(H+l_{iz}\right)/\left(\cos\left(\beta_{S}^{\prime }\right)\cos\left(\gamma_{S}^{\prime }\right)\right)\\ {}^{B_{Si}}P_{Six}=-k\;\cdot \;\sin\left(\beta_{S}^{\prime }\right)\\ {}^{B_{Si}}P_{Siy}=k\;\cdot \;\cos\left(\beta_{S}^{\prime }\right)\sin\left(\gamma_{S}^{\prime }\right) \end{array}\right.$$

Due to the existing distance $L_{1}$ between the hypothetical base coordinate and the plane of the parallelogram mechanism of the leg, ${}^{B_{Si}}P_{Siy}$ needs to be updated according to the actual robot structure, as shown in Equation (31):

(31) $${}^{B_{Si}}P_{Siy}=k\;\cdot \;\cos\left(\beta_{S}^{\prime }\right)\sin\left(\gamma_{S}^{\prime }\right)+L_{1}$$

Under the hypothetical posture of the robot, the robot body coordinate *C* is the same as the slope coordinate *S*. Therefore, based on Equation (3), the hypothetical foot position of leg *i* in the slope coordinate *S* can be obtained, as shown in Equation (32):

(32) $$\left\{\begin{array}{c} {}^{S}P_{Six}={}^{B_{Si}}P_{Six}+l_{ix}\\ {}^{S}P_{Siy}={}^{B_{Si}}P_{Siy}+l_{iy}\\ {}^{S}P_{Siz}={}^{B_{Si}}P_{Siz}+l_{iz} \end{array}\right.$$

where ${}^{S}P_{Si}={\left[{}^{S}P_{Six},{}^{S}P_{Siy},{}^{S}P_{Siz}\right]}^{T}$ represents the hypothetical foot position vector of leg *i* in the slope coordinate *S* when the robot is standing still on the slope.

To maintain as high stability as possible at any time during walking, ${}^{S}P_{Si}$ should be kept in the middle position of a step. Therefore, based on the robot step lengths and steering angle set by the robot users, the landing foot position of the swing leg *i* can be obtained, as shown in Equation (33):

(33) $$\left\{\begin{array}{c} {}^{S}P_{ix3}={}^{S}P_{Six}\cos\left(S_{\theta }/2\right)-{}^{S}P_{Siy}\sin\left(S_{\theta }/2\right)+S_{x}/2\\ {}^{S}P_{iy3}={}^{S}P_{Six}\sin\left(S_{\theta }/2\right)+{}^{S}P_{Siy}\cos\left(S_{\theta }/2\right)+S_{y}/2\\ {}^{S}P_{iz3}={}^{S}P_{Siz} \end{array}\right.$$

where ${}^{S}P_{i3}={\left[{}^{S}P_{ix3},{}^{S}P_{iy3},{}^{S}P_{iz3}\right]}^{T}$ represents the landing foot position vector of the swing leg *i* in coordinate *S*. $S_{x}$ and $S_{y}$ represent the step lengths along the *x*- and *y*-directions of the slope coordinate *S* which are set by the robot users during walking. $S_{\theta }$ represents the steering angle of the robot set by the robot users. For a straight walking process, $S_{y}$ and $S_{\theta }$ are set to zero.

Based on the landing foot position of the swing leg *i* calculated, and the walking parameters set by the robot users, the landing foot velocity of the swing leg *i* can be obtained, as shown in Equation (34):

(34) $$\left\{\begin{array}{c} {}^{S}vP_{ix3}=-v_{x}+{}^{S}P_{iy3}\;\cdot \;\omega \\ {}^{S}vP_{iy3}=-v_{y}-{}^{S}P_{ix3}\;\cdot \;\omega \\ {}^{S}vP_{iz3}=0 \end{array}\right.$$

with

(35) $$\left\{\begin{array}{c} v_{x}=S_{x}/\left(\lambda \;\cdot \;T\right)\\ v_{y}=S_{y}/\left(\lambda \;\cdot \;T\right)\\ \omega =S_{\theta }/\left(\lambda \;\cdot \;T\right) \end{array}\right.$$

where ${}^{S}vP_{i3}={\left[{}^{S}vP_{ix3},{}^{S}vP_{iy3},{}^{S}vP_{iz3}\right]}^{T}$ represents the landing foot velocity vector of the swing leg *i* in coordinate *S*. $v={\left[v_{x},v_{y},0\right]}^{T}$ represents the translational velocity vector of the robot body in coordinate *S*. ω represents the angular velocity of the robot body.

The landing foot acceleration of the swing leg *i* can be calculated using the differential method, as shown in Equation (36):

(36) $${}^{S}aP_{i3}=\frac{d}{dt}{}^{S}vP_{i3}$$

For a swing leg, the step height *h* is usually set by the robot users before walking. Then, based on the step height, the starting foot position and landing foot position of the swing leg *i* calculated, the middle foot position can be obtained, as shown in Equation (37):

(37) $$\left\{\begin{array}{c} {}^{S}P_{ix2}=\left({}^{S}P_{ix1}+{}^{S}P_{ix3}\right)/2\\ {}^{S}P_{iy2}=\left({}^{S}P_{iy1}+{}^{S}P_{iy3}\right)/2\\ {}^{S}P_{iz2}=-H+h \end{array}\right.$$

where ${}^{S}P_{i2}={\left[{}^{S}P_{ix2},{}^{S}P_{iy2},{}^{S}P_{iz2}\right]}^{T}$ represents the middle foot position vector of the swing leg *i* in coordinate *S*.

Based on ${}^{S}P_{i1}$ from Equation (26), ${}^{S}vP_{i1}$ and ${}^{S}aP_{i1}$ from Equation (27), ${}^{S}P_{i3}$ from Equation (33), ${}^{S}vP_{i3}$ from Equation (34), ${}^{S}aP_{i3}$ from Equation (36), and ${}^{S}P_{i2}$ from Equation (37), the unknown polynomial parameters in Equation (25) can be calculated. Based on the polynomial parameters calculated, the swing leg trajectory of leg *i* in coordinate *S* can be finally obtained using Equation (22). Then, considering the actual body attitude, the swing leg trajectory of leg *i* in the body coordinate *C* can be easily obtained, as shown in Equation (38):

(38) $${}^{C}P_{i}={}_{C}^{W}{R^{\prime }}^{T}\;\cdot \;{}_{S}^{W}R^{\prime }\;\cdot \;{}^{S}P_{i}$$

By using the inverse kinematics model shown in Equation (2), the joint trajectories which satisfy the swing leg trajectory of leg *i* designed can be calculated. Then, with the motions of the joints, the actual swing leg trajectory of leg *i* with both the body attitude angles and the terrain attitude angles considered can be finally generated.

#### 3.2.2. Support Leg Trajectory Generation

For the balance locomotion generation of a legged creature, the destination, moving velocity, and body posture may be firstly determined. Then, the motions of the support legs to keep the body balance are generated automatically by the central nervous system. Namely, to generate the motions of the support legs, the body displacement trajectory, and the body attitude trajectories should be defined first. The attitude trajectories can be planned through the balance attitude trajectory planning process, so the critical point to generate the support leg trajectory is to obtain the body displacement trajectory.

Based on the gait parameters set by the robot users, the body moving velocities along different directions of the slope coordinate *S* can be obtained through the computation of Equation (35). Then, based on the actual attitude angles of the slope, the global moving velocity vector ${}^{W}V$ of the robot can be obtained, as shown in Equation (39):

(39) $$\left[\begin{array}{c} {}^{W}V_{x}\\ {}^{W}V_{y}\\ {}^{W}V_{z} \end{array}\right]={}_{S}^{W}R^{\prime }\;\cdot \;\left[\begin{array}{c} v_{x}\\ v_{y}\\ 0 \end{array}\right]$$

During every attitude adjustment cycle, the global body displacement trajectory of the robot can be calculated by integrating the global moving velocity in the adjustment time, as shown in Equation (40):

(40) $${}^{W}Q=\int_{0}^{t_{r}}{}^{W}Vdt$$

where ${}^{W}Q={\left[{}^{W}Q_{x},{}^{W}Q_{y},{}^{W}Q_{z}\right]}^{T}$ represents the global body displacement vector of the robot during one attitude adjustment cycle.

During every attitude adjustment cycle, the robot body and the support legs together can be treated as a parallel mechanism. At the beginning time of an attitude adjustment cycle, namely at time $t_{r}=0$, the origin of the body coordinate *C* is defined as the origin of the global coordinate *W*. Therefore, based on the initial body attitude angles at the time $t_{r}=0$ obtained by the IMU feedback signals, the foot position of the support leg *i* at time $t_{r}=0$ in the global coordinate *W* can be obtained, as shown in Equation (41):

(41) $$\left[\begin{array}{c} {}^{W}{P_{i}}_{x0}\\ {}^{W}{P_{i}}_{y0}\\ {}^{W}{P_{i}}_{z0} \end{array}\right]={}_{C}^{W}R^{\prime }\left[\begin{array}{c} {}^{C}{P_{i}}_{x0}\\ {}^{C}{P_{i}}_{y0}\\ {}^{C}{P_{i}}_{z0} \end{array}\right]$$

where ${}^{W}P_{i0}={\left[{}^{W}P_{ix0},{}^{W}P_{iy0},{}^{W}P_{iz0}\right]}^{T}$ represents the foot position of leg *i* in the global coordinate at the time $t_{r}=0$.

As the robot can be treated as a parallel mechanism, ${}^{W}P_{i0}$ can be treated as the fixed platform position of the parallel mechanism. Therefore, during one attitude adjustment cycle, the desired support trajectory of leg *i* to ensure the walking balance of the robot in the body coordinate *C* can be obtained based on the inverse kinematics computation of the parallel mechanism, the planned attitude trajectories, and the body displacement trajectory, as shown in Equation (42):

(42) $$\left[\begin{array}{c} {}^{C}{P_{i}}_{x}\\ {}^{C}{P_{i}}_{y}\\ {}^{C}{P_{i}}_{z}\\ 1 \end{array}\right]=\left[\begin{array}{cccc} & {}_{C}^{W}R_{t_{r}}^{T} & & -{}_{C}^{W}R_{t_{r}}^{T}\;\cdot \;{}^{W}Q\\ 0 & 0 & 0 & 1 \end{array}\right]\left[\begin{array}{c} {}^{W}{P_{i}}_{x0}\\ {}^{W}{P_{i}}_{y0}\\ {}^{W}{P_{i}}_{z0}\\ 1 \end{array}\right]$$

where ${}_{C}^{W}R_{t_{r}}$ represents the the planned rotation transformation matrix of the body coordinate *C* with respect to the global coordinate *W*. ${}_{C}^{W}R_{t_{r}}$ has the same form as $R_{w}$ shown in Equation (10), and consists of the balance yaw, pitch, and roll trajectories planned in Equation (4).

It can be seen from Equation (42) that the support trajectory of leg *i* is automatically generated according to the planned balance body attitude trajectories. Then, based on Equations (2) and (3), the joint trajectories of leg *i* can be computed.

During hexapod locomotion, the body attitude trajectories are continually planned according to the motion state of the robot to keep the body balance. Therefore, the support leg trajectories of the robot are continuously updated to adjust the body deviations. Through this process, the body attitude trajectories during walking will be optimized and the walking balance of the robot will be ensured.

## 4. Experiments and Result Discussion

### 4.1. General Introduction of the Experiments

To verify the effectiveness of the attitude trajectory optimization method in ensuring balance hexapod locomotion of the large-size hexapod robot, two typical terrain walking experiments were carried out on the real large-size robot. First, an artificial soft terrain walking experiment was carried out. Walking performances under different balance control methods were compared to show the advantages of the attitude trajectory optimization method proposed. Second, a natural soft terrain walking experiment was carried out to show the applicability of the attitude trajectory optimization method in improving the hexapod walking performance in the real outdoor environment. In all these experiments, only the triangle gait form with three legs supporting the robot body at the same time was employed due to its sensitivity to the changes of the terrain.

**Figure 9:**
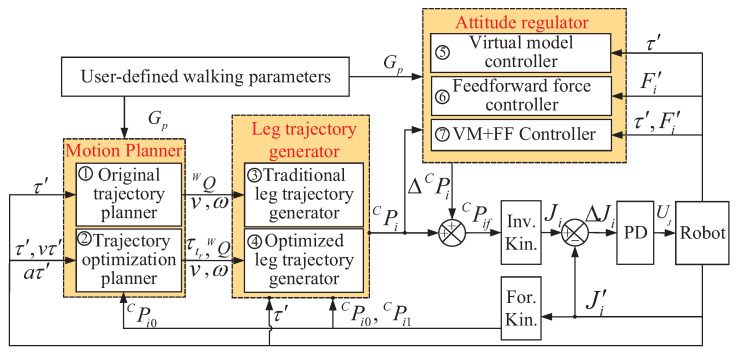
The brief structure of the control scheme of the robot.

To better demonstrate the advantages of the attitude trajectory optimization method proposed, some typical control schemes were employed as comparisons during the experiment processes. Figure 9 shows the brief structure of the control scheme of the hexapod robot. The unknown parameters in Figure 9 which are not mentioned in the paper are explained in Table 1.

It can be seen from Figure 9 that, in the control scheme of the hexapod robot, two options can be selected to be the motion planner. Two options can be selected to be the leg trajectory generator while three options can be selected to be the attitude regulator. To ensure a basic hexapod walking process, the motion planner and the leg trajectory generator must be selected to compose a basic control scheme, but the attitude regulator may not be necessary. Through the combinations of the different options, several typical control schemes were formed and employed in the experiments to show the control performances of different control methods. The brief introductions of the control schemes are as follows:

**1. Option 1**+Option 3: when option 1 and option 3 are selected, the control scheme is named as **WO**, which refers to the original walking control scheme without attitude adjustment. The robot can only ensure a basic motion process without any regulation of the walking attitude when this control scheme is employed. In addition, the swing leg trajectory is designed in the body coordinate *C* without considering the terrain attitude.

**2. Option 2+Option 4:** when option 2 and option 4 are selected, the control scheme is named as **WT**, which refers to walking with the attitude trajectory optimization method proposed. When this control scheme is employed, the walking attitude trajectories can be optimized under the function of the attitude trajectory optimization method proposed.

**3. Option 1+Option 3+Option 5:** when option 1, option 3, and option 5 are selected, the control scheme is named as **WV**, which refers to walking with the virtual model controller. When this control scheme is employed, the walking attitude angles can be regulated under the function of the virtual model. The swing leg trajectory is designed in the body coordinate *C* without considering the terrain attitude. The virtual model employed can be found in [29], Equations (2)–(16).

**4. Option 1+Option 3+Option 6:** when option 1, option 3, and option 6 are selected, the control scheme is named as **WF**, which refers to walking with the feedforward force controller. When this control scheme is employed, the contact foot forces can be regulated through a feedforward way to reduce walking attitude fluctuations. The swing leg trajectory is designed in the body coordinate *C* without considering the terrain attitude. This control scheme is as same as the control scheme proposed in the authors’ previous work [23].

**5. Option 1+Option 3+Option 7:** when option 1, option 3, and option 7 are selected, the control scheme is named as **WFV**, which refers to walking with the controller that combines the feedforward force control method and the virtual model control method. When this control scheme is employed, both the contact foot forces and the walking attitude angles can be regulated. However, the swing leg trajectory is designed in the body coordinate *C* without considering the terrain attitude.

**6. Option 2+Option 4+Option 6:** when option 2, option 4, and option 6 are selected, the control scheme is named as **WFT**, which refers to walking with the controller that combines the feedforward force control method and the attitude trajectory optimization method proposed. When this control scheme is employed, both the contact foot forces and the walking attitude angles can be regulated. The swing leg trajectory is designed with both the robot attitude and the terrain attitude considered.

During the experiment processes, WO, WV, and WF were employed to make comparisons in walking performance with WT, namely the control scheme using the attitude trajectory optimization method proposed. Through this way, the advantages of the method proposed can be easily demonstrated. WFV was employed to make a comparison with WFT to demonstrate that the optimization method proposed can be easily combined with other control methods to further improve the walking balance of the hexapod robot.

### 4.2. The Artificial Soft Terrain Walking Experiment

The real outdoor terrains which legged robots walk on are usually soft and uneven. For a legged robot, walking balance on uneven terrain with soft soil must be ensured if good environmental adaptability is wanted to be achieved. To verify the effectiveness of the attitude trajectory optimization method proposed in ensuring the walking balance of the real large-size hexapod robot on uneven soft terrains, an artificial soft terrain walking experiment imitating the soft terrain walking process of the large-size hexapod robot was carried out.

**Figure 10:**
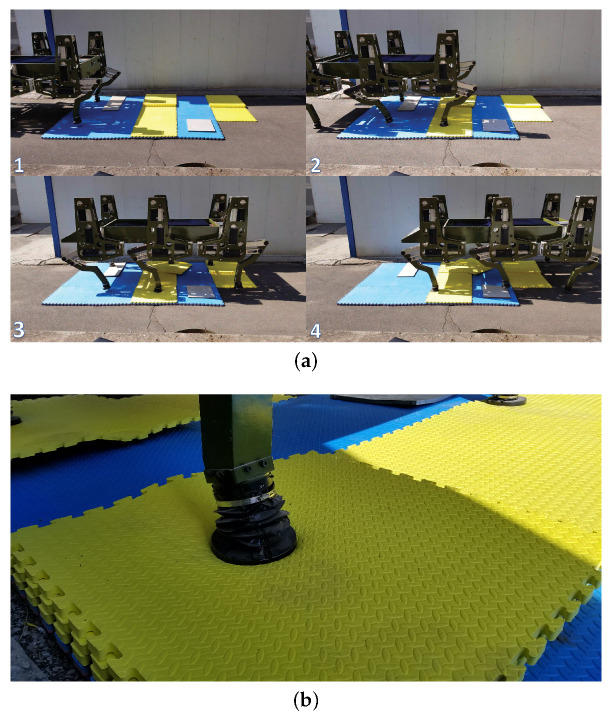
The snapshots of the artificial soft terrain walking experiment: (**a**) the artificial soft terrain walking process and (**b**) the deformation of the artificial soft terrain.

The walking parameters employed in the experiment is shown in Table 2. The artificial soft terrain walking process of the large-size hexapod robot is shown in Figure 10a. The artificial soft terrain was mainly constructed of EPE plates. The size of each EPE plate is 1 m × 1 m × 0.02 m. Two layers of the blue EPE plates were used to construct the basement of the terrain. Several yellow EPE plates and two plywood plates were randomly placed on and beside the terrain basement to simulate the uneven characteristics of the natural terrains. The thickest part of the terrain was constructed of five layers of the EPE plates. Obvious deformation of the EPE plate being compressed could be seen during walking, as shown in Figure 10b. Therefore, the artificial soft terrain can pose a great challenge to the walking balance of the robot.

**Figure 11:**
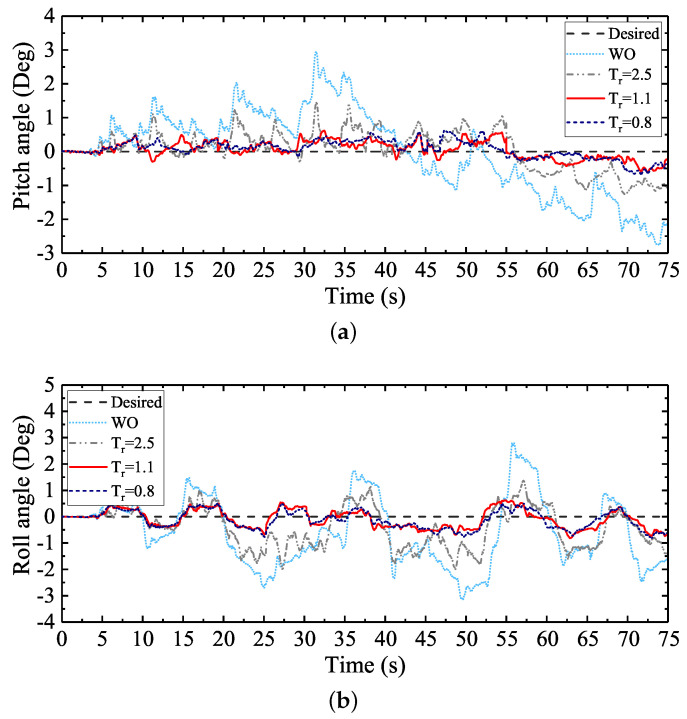
The attitude regulation performances of the pitch and roll angles with different $T_{r}$: (**a**) the body pitch angle comparison and (**b**) the body roll angle comparison.

According to the theoretical design of the attitude trajectory optimization method proposed, the attitude deviation adjustment time $T_{r}$ needs to be defined by users if a good attitude regulation performance is wanted to be ensured. Figure 11 shows the regulation performances of the pitch and roll angles of WT with different $T_{r}$ defined. The max absolute tracking errors and the standard deviations of the attitude angle regulation processes are shown in Table 3, where Max. Abs. TE represents the max absolute tracking error, and Std. Deviation represents the standard deviation.

Ideally, during the walking process, both the desired pitch and roll angles should be zero. However, it can be seen from Figure 11 that the attitude angles fluctuated severely when WO was employed. In other words, the walking balance of the large-size hexapod robot was severely challenged by the terrain condition and the large inertia of the robot if no attitude regulation method was employed. During a hexapod walking process, small attitude angle deviations will gradually accumulate if no regulation method is employed. The large mass and inertia of the robot will aggravate the accumulations of the deviations in a short time, and the walking balance will be severely challenged.

Compared with WO, the walking balance of the hexapod robot was significantly improved when the control scheme WT was employed. Based on the gait parameters shown in Table 2 and Equation (19), it can be analyzed that, during one gait cycle in the experiment, the supporting time was 5 s. According to Equation (19), the deviation adjustment time $T_{r}$ should be within 5 s. When $T_{r}$ was defined to 2.5 s, the attitude angle fluctuations were obviously reduced. Compared with WO, the max absolute tracking errors of the pitch and roll angles were reduced by 51% and 36%, respectively. The standard deviations of the pitch and roll angles were reduced by 54% and 41%, respectively. When $T_{r}$ was defined to 1.1 s, the max absolute tracking errors of the pitch and roll angles were reduced by 79% and 74%, respectively. The standard deviations of the pitch and roll angles were reduced by 79% and 76%, respectively. When $T_{r}$ was defined to 0.8 s, the max absolute tracking errors of the pitch and roll angles were reduced by 77% and 74%, respectively. The standard deviations of the pitch and roll angles were reduced by 79% and 74%, respectively.

It can be analyzed from the attitude regulation results that, with the decrease of $T_{r}$, the attitude regulation process was accelerated, and better walking attitude angles were ensured. Nevertheless, the attitude deviations could not be completely eliminated with the change of $T_{r}$. Actually, the attitude regulation performances of $T_{r}$ = 1.1 s and $T_{r}$ = 0.8 s were almost the same. The little attitude regulation difference between them may be caused by the little changes of experimental conditions (e.g., the little initial location changes of the robot). For robot users, $T_{r}$ should be defined according to the actual regulation performances, just like the conventional PID parameter adjustment process.

**Figure 12:**
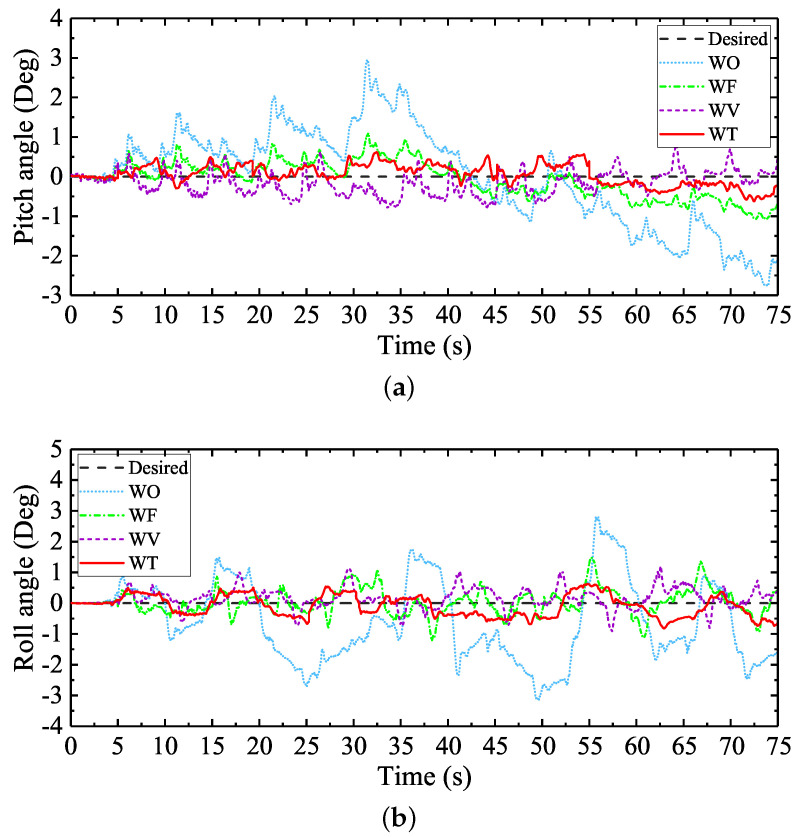
The attitude regulation performances of the pitch and roll angles under different control schemes: (**a**) the body pitch angle comparison and (**b**) the body roll angle comparison.

As introduced in Section 4.1, to better demonstrate the advantages of the attitude trajectory optimization method proposed in ensuring balance hexapod locomotion, different typical control schemes were employed as comparisons during the experiment. Figure 12 shows the regulation performances of the pitch and roll angles under different control schemes. The max absolute tracking errors and the standard deviations of the attitude angle regulation processes are shown in Table 4. According to the balance walking control performances under different $T_{r}$, $T_{r}$ was finally defined to be 1.1 s when WT was employed.

When WF was employed, the attitude angle fluctuations were obviously reduced due to the regulation of the contact foot forces. This phenomenon verified that the contact foot force is a key factor which can affect the walking balance of the large-size hexapod robot. However, compared with the control performances of WV and WT, the max absolute tracking errors and the standard deviations of the attitude angles with WF employed were larger. The feedforward foot force control method cannot best ensure the walking balance of the robot only through the regulation of the foot forces.

Compared with the control performance of WF, the attitude angle fluctuations were further reduced when WV and WT were employed. However, it can be seen from Figure 12 that, when WV was employed, the walking attitude angles changed suddenly at each time when the swing legs switched into the support legs, and larger attitude tracking errors occurred. This may be caused by the sudden changes of the foot position compensation values brought by the virtual model. According to the attitude regulation results shown in Table 4, compared with WV, the max absolute tracking errors and the standard deviations of the attitude angles with WT employed were smaller. The tracking error reductions of the attitude angles verified that the proposed attitude trajectory optimization method could suppress the sudden attitude angle changes caused by the control features of the virtual model control method. Namely, the body attitude adjustment process is more stable. Considering the overall control performance and the simplicity of WT, the attitude trajectory optimization method proposed is better to be employed in ensuring the walking balance of the large-size hexapod robot.

**Figure 13:**
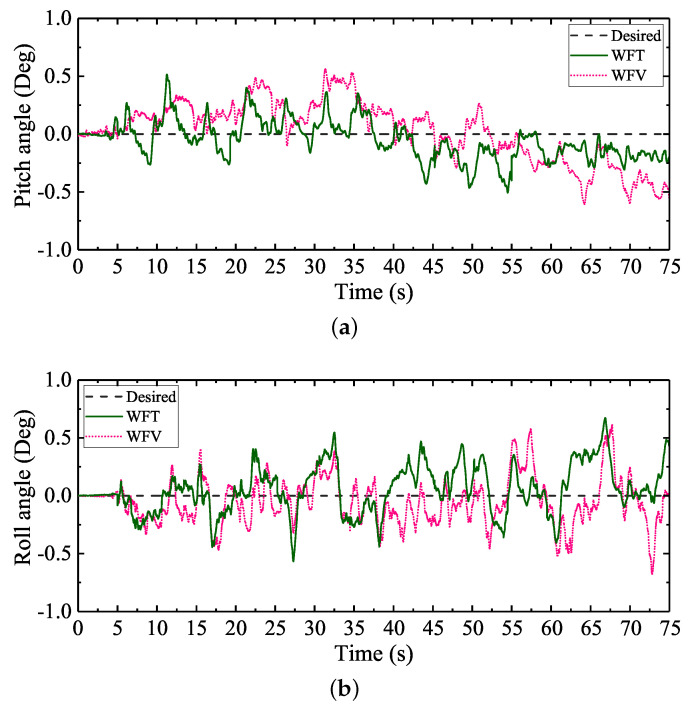
The attitude regulation performances of the pitch and roll angles under WFT and WFV: (**a**) the body pitch angle comparison and (**b**) the body roll angle comparison.

Since the foot force is a key factor which can impact the walking balance of a legged robot, some scholars made efforts to combine the virtual model control method with the feedforward foot force control method to further improve the walking balance of legged robots, like reference [29]. To further verify the fact that the attitude trajectory optimization method proposed can be easily combined with other control methods to ensure better walking balance of the large-size hexapod robot, the control scheme WFT which combined the feedforward foot force control method and the attitude trajectory optimization method proposed was employed to make comparisons with the control scheme WFV which combined the feedforward foot force control method and the virtual model control method. Figure 13 shows the regulation performances of the pitch and roll angles under the two control schemes. The max absolute tracking errors and the standard deviations of the attitude angle regulation processes are shown in the last two rows of Table 4.

It can be seen from Figure 13 and Table 4 that, compared with WF, WV, and WT, the attitude angle fluctuations were further reduced when WFV and WFT were employed. The artificial soft terrain in this experiment was not ideally flat. The walking attitude deviations were mainly caused by the local profile changes on the terrain. According to the theoretically design, when WT was employed, the control system kept planning new body attitude trajectories based on the walking attitude deviations, and generated new support leg trajectories to regulate the walking balance of the robot. When WV was employed, the control system kept computing the foot position compensation values based on the attitude deviations, and regulated the motions of the support legs to reduce the attitude deviations. Namely, for both the virtual model control method and the attitude trajectory optimization method, the motions of the legs could not directly respond to the local profile changes on the terrain. Only when the walking attitude deviations occurred did the two control schemes begin to work. However, when the feedforward foot force control method was combined with the two control methods, due to the foot impedance model in the feedforward foot force control method, the foot position compensation values were directly computed based on the foot force deviations. Under this function, the legs of the robot would rise or stretch directly to adapt to the local profile changes on the terrain. Then, with the control processes of the virtual model control method and the attitude trajectory optimization method, the walking attitude fluctuations were further reduced.

The attitude regulation performance of WFT was a little bit better than the regulation performance of WFV. Both of the two control schemes can ensure balance hexapod locomotion. However, compared with WFV, the structure of WFT is more simple. In addition, without considering the leg impedance parameters in the feedforward foot force control method, only $T_{r}$ in WFT needs to be defined by the robot users to achieve good control performance. In contrast to WFT, achieving the same control performance, the stiffness and damping coefficients of the virtual model in WFV must be well designed by the robot users. Considering the simplicity of the control scheme structure and the convenience of the parameter adjusting process, the attitude trajectory optimization method proposed is easier to be employed to design a balance walking control system of a legged robot.

### 4.3. The Natural Soft Terrain Walking Experiment

Through the artificial soft terrain walking experiment, the effectiveness of the attitude trajectory optimization method proposed in ensuring the walking balance of the real large-size hexapod robot has been verified. Nevertheless, the real outdoor terrains which legged robots usually walk on are not as the same as the artificial soft terrain employed. The deformation of the artificial soft terrain constructed of the EPE plates is elastic. However, for natural soft terrains, the deformation is usually plastic. The plastic deformation of a natural soft terrain may cause more obvious foot sinkage and severely challenge the walking balance of a legged robot. In addition, a natural uneven terrain is not ideally flat. The unpredictable slope may further lead to an unbalance walking process of a legged robot. To further verify the practical feasibility of the attitude trajectory optimization method proposed in ensuring the walking balance of the large-size hexapod robot on real outdoor terrains, a natural soft terrain walking experiment was carried out. The walking parameters employed during the experiment is shown in Table 5.

**Figure 14:**
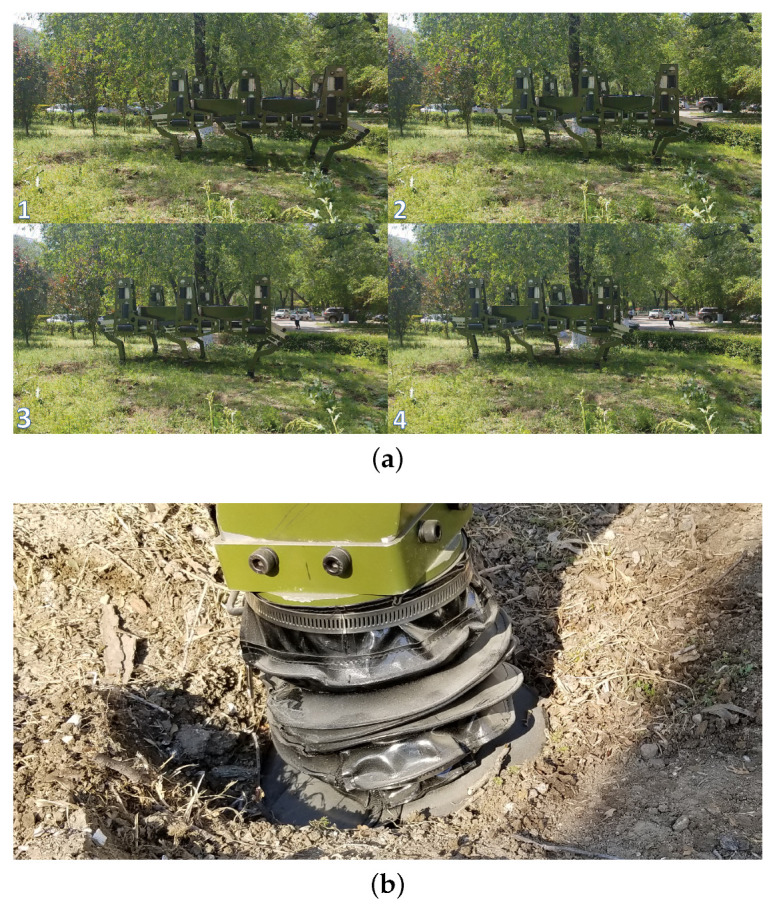
The snapshots of the natural soft terrain walking experiment: (**a**) the natural soft terrain walking process and (**b**) the foot sinkage caused by the deformation of the natural soft terrain.

**Figure 15:**
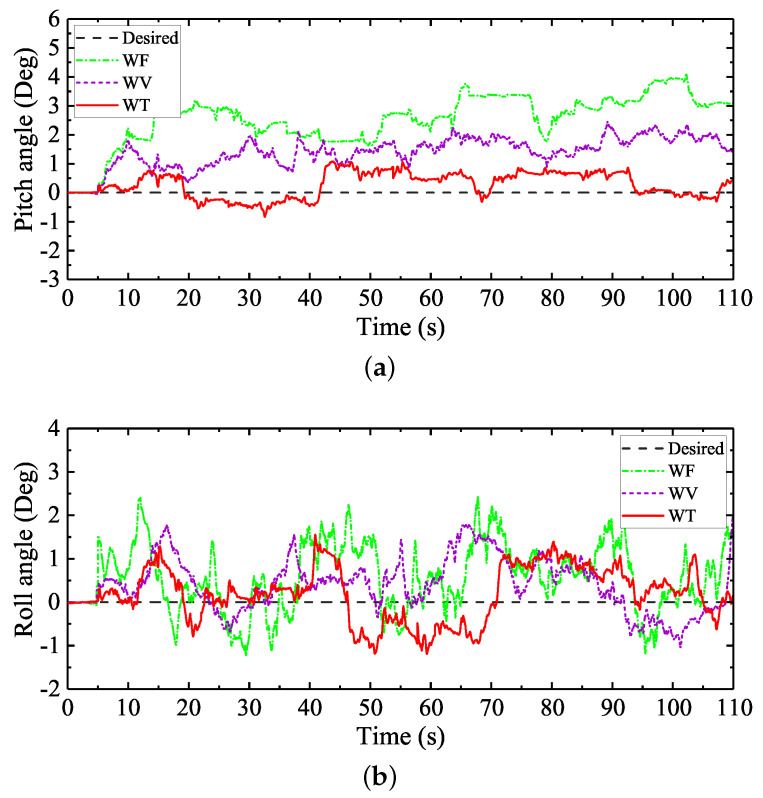
The attitude regulation performances of the pitch and roll angles under different control schemes: (**a**) the body pitch angle comparison and (**b**) the body roll angle comparison.

The natural soft terrain walking process of the large-size hexapod robot is shown in Figure 14a. The terrain soil is soft and has a certain small slope. During the walking experiment, obvious foot sinkage caused by the plastic deformation of the terrain soil was found, as shown in Figure 14b. To ensure the walking stability of the large-size hexapod robot on the slope, the desired pitch and roll angles were set to be zero, and the control scheme WO with no regulation of the attitude angles was not employed in this experiment. Figure 15 shows the regulation performances of the pitch and roll angles under WF, WV, and WT. The max absolute tracking errors and the standard deviations of the attitude regulation processes are shown in Table 6. During the experiment process, $T_{r}$ was defined to be 1.1 s when WT was employed.

Comparing the attitude regulation results shown in Table 4 and Table 6, it can be analyzed that the natural soft terrain conditions brought more severe challenges on the walking balance of the large-size hexapod robot. From Figure 15a, it can be seen that, when WF and WV were employed, the actual pitch angles under WF and WV were far beyond the desired value. This phenomenon was mainly caused by the small unpredictable slope on the terrain. When WF and WV were employed, the swing leg trajectories designed in the body coordinate *C* without considering the terrain attitude would cause the early landings of some swing legs, especially the front swing legs. The early landing swing legs would keep swinging to reach the unchangeable landing positions, like the situation shown in Figure 6. Due to this fact, the robot body would be lifted. When WF was employed, the robot body was forced to be parallel to the slope surface. When WV was employed, although the body attitude tracking performance was better than WF, the body attitude adjustment process under the function of the virtual model controller was still obstructed by the motions of the early landing swing legs. Complete attitude tracking was still not satisfied.

Different from the control performances of WF and WV, when WT was employed, the desired attitude angles were almost tracked. Compared with the attitude regulation results of WF and WV, the max absolute tracking errors and the standard deviations of the pitch and roll angles were obviously reduced when WT was employed, as shown in Table 6. The obvious reductions of the attitude fluctuations indicated that the walking balance of the large-size hexapod robot was significantly improved under the function of the attitude trajectory optimization method proposed.

**Figure 16:**
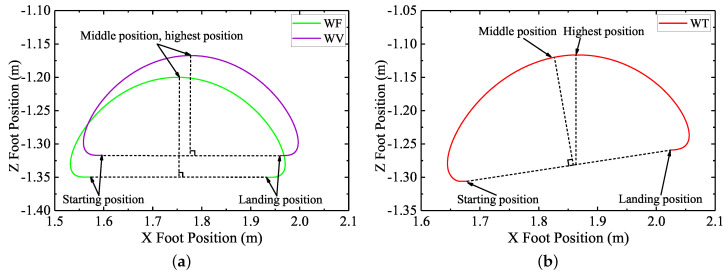
The planned swing trajectories of leg 1 in the robot body coordinate *C* during one gait cycle: (**a**) the planned swing trajectories of leg 1 with WF and WV employed and (**b**) the planned swing trajectory of leg 1 with WT employed.

Figure 16 shows the planned swing trajectories of leg 1 in the robot body coordinate *C* during a same gait cycle with different control schemes employed. It can be seen from Figure 16a that, when WF was employed, due to the traditional swing leg trajectory design process with no terrain attitude angles considered, the planned landing position of leg 1 along the *z*-direction of the body coordinate *C* was as same as the starting position of leg 1 along the *z*-direction of the body coordinate *C*. At the beginning of the walking process, the initial body pitch angle was set to be zero, as shown in Figure 15a. During the first gait cycle because the body is not parallel to the slope, and the landing foot position of leg 1 along the *z*-direction of the body coordinate *C* is the same as the starting foot position, the early landing of leg 1 occurred. As leg 1 continued to move towards the target landing foot position, the robot body was lifted and forced to be parallel to the slope. Due to the control features of the feedforward foot force control method, the desired pitch angle would not be tracked. The robot body kept being parallel to the slope during walking.

Similar to the swing trajectory of leg 1 with WF employed, when WV was employed, the landing foot position of leg 1 along the *z*-direction of the body coordinate *C* was the same as the starting foot position. Different from the control features of WF, the robot would try to track the desired body attitude under the function of the virtual model control method. Nevertheless, due to the interference of the early landing swing legs’ movement, the body attitude adjustment would be obstructed. Then, under the combined action of the body attitude adjustment process and the motion behavior of the early landing swing legs, the actual attitude of the robot was neither parallel to the slope nor could it track the desired attitude. This is why the starting foot position of leg 1 with WV employed was not the same as the starting foot position of leg 1 with WF employed, as shown in Figure 16a.

In sharp contrast to the swing trajectories of leg 1 with WF and WV employed, the swing trajectory of leg 1 with WT employed during walking was different, as shown in Figure 16b. Due to the swing trajectory generation process of the attitude trajectory optimization method proposed with the actual body and terrain attitude angles considered, the landing foot position of leg 1 along the *z*-direction of the body coordinate *C* was not the same as the starting foot position. In addition, through the traditional swing trajectory design process of WF and WV, the middle position and the highest position of the swing trajectory of leg 1 were the same. Namely, the shape of the swing leg trajectory during every gait cycle when WF and WV were employed would always be the same. However, when WT was employed, the middle position and the highest position of the swing trajectory of leg 1 were not the same. During the walking process of the large-size hexapod robot with WT employed, the trajectory of a swing leg would keep changing to adapt to the changes of the actual terrain attitude angles and the actual robot body attitude angles. Through this way, the influence of the swing leg movements on the body attitude adjustment process was reduced. Then, together with the movements of the support legs generated from the balance attitude trajectories planned, the actual walking attitude trajectories of the large-size hexapod robot with WT employed were optimized, and the walking balance of the large-size hexapod robot was ensured.

**Figure 17:**
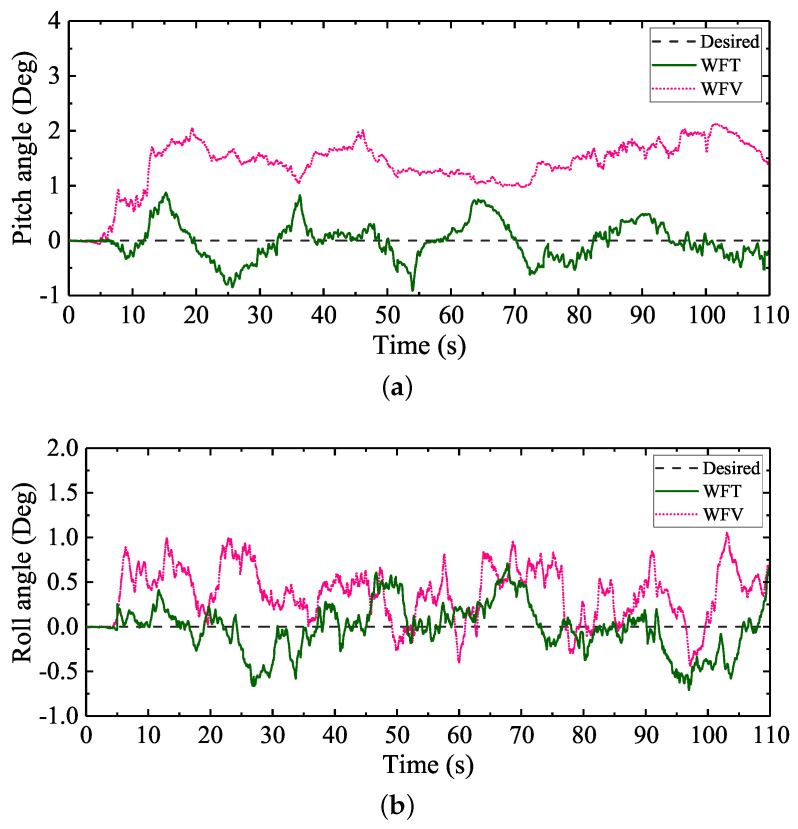
The attitude regulation performances of the pitch and roll angles under WFT and WFV: (**a**) the body pitch angle comparison and (**b**) the body roll angle comparison.

Like the artificial soft terrain walking experiment, to further improve the walking balance of the large-size hexapod robot, WFT and WFV were employed in the natural soft terrain walking experiment. The attitude regulation comparisons between the two control schemes are shown in Figure 17. The max absolute tracking errors and the standard deviations of the attitude angles under WFT and WFV are shown in the last two rows of Table 6.

It can be seen from Figure 17a that, when the feedforward foot force control method was combined with the virtual model control method, namely when WFV was employed, the pitch angle was still far beyond the desired value. Compared with the attitude regulation results of WF and WV shown in Table 6, the max absolute tracking errors and the standard deviations of the pitch and roll angles with WFV employed were reduced, namely the walking attitude fluctuations were reduced. However, due to the interference of the swing leg movements on the body attitude adjustment discussed above, complete attitude tracking was still not satisfied. In contrast to the control performance of WFV, when WFT was employed, the desired body attitude angles were almost tracked. Compared with the attitude regulation results of WT shown in Table 6, when the feedforward foot force control method was combined with the attitude trajectory optimization method proposed, the attitude fluctuations were further reduced. It can be analyzed from the overall experiment results of the natural soft terrain walking experiment that, compared with the commonly used balance walking control methods for legged robots, the attitude trajectory optimization method proposed in this paper can better improve the walking balance of a slope-climbing legged robot. Furthermore, the attitude trajectory optimization method proposed in this paper can simplify the control system design and can be easily combined with other control methods to further ensure the walking balance of a legged robot.

## 5. Conclusions and Further Works

In this paper, the importance of the walking balance control of a large-size hexapod robot has been demonstrated. The main theoretical contribution of this paper is the proposal of a simple attitude trajectory optimization method which can ensure the walking balance of the large-size hexapod robot on different terrains. The attitude trajectory optimization method is mainly designed with two stages: the balance attitude trajectory planning stage and the leg trajectory generation stage. During walking, the balance attitude trajectory considering different walking constraints is firstly planned via high order polynomial interpolation. Then, the swing leg trajectory is well designed with the terrain attitude angles considered. The support leg trajectory is automatically generated based on the balance attitude trajectories planned to ensure the walking balance of the large-size hexapod robot. To verify the effectiveness of the attitude trajectory optimization method proposed in ensuring the walking balance of the large-size hexapod robot, different outdoor terrain walking experiments have been carried out on the real large-size hexapod robot. The experiment results clearly demonstrate that, compared with the currently developed balance walking control methods for legged robots, the attitude trajectory optimization method proposed in this paper can better improve the walking balance of a hexapod robot and is easier to be combined with other control methods to further optimize the walking attitude of a hexapod robot. In addition, it is easier to be employed to simplify the control system design of a hexapod robot. Considering the advantages of the control method proposed, more applications of the attitude trajectory optimization method proposed on different legged robots can be expected.

Limited by the current experiment conditions and the inconvenient transportation of the large-size hexapod robot, the walking experiments of the large-size hexapod robot were carried out on the terrains with unchanged physical properties. Nevertheless, on a more challenging terrain with complex physical properties, the leg trajectories may be badly affected by the complex physical properties of the terrain. Then, the walking balance of the large-size hexapod robot may be further influenced. In addition, the slope angle in the natural soft terrain walking experiment is small. However, in a more challenging environment, the slope angle may be larger. In the authors’ recently published work [36], a vision-based framework to infer the physical properties of the terrain is proposed. Furthermore, the framework in [36] will be introduced into the control system of the large-size hexapod robot, and the leg trajectory generation process in the attitude trajectory optimization method proposed in this paper will be updated to cooperate with the function of the framework to further improve the walking balance of the large-size hexapod robot on more challenging terrains. More experiments of the large-size hexapod robot walking on challenging terrains will be carried out in the future to validate the effectiveness of the control method proposed, especially the slope-climbing experiment with a large slope angle.

## Figures and Tables

**Table 1:** Unknown parameters of the control scheme.

Parameter	Quantity
$G_{p}$	The general symbol used to represent the user-defined robot walking parameters,
	including the gait parameters, desired attitude angles, etc
$\tau^{\prime }$	The general symbol used to represent the actual attitude angles of the robot
$v\tau^{\prime }$	The general symbol used to represent the actual attitude angle velocities of the robot
$a\tau^{\prime }$	The general symbol representing the actual attitude angle accelerations of the robot
$F_{i}^{\prime }$	The actual foot force vector of leg *i*
$J_{i}^{\prime }$	The actual joint position vector of leg *i*
$\Delta J_{i}$	The joint position deviation
$U_{J}$	The control quantity of the joint motor
$\Delta {}^{C}P_{i}$	The foot position compensation value of leg *i*
${}^{C}P_{if}$	The final desired foot position of leg *i*

**Table 2:** Walking parameters.

Parameter	Quantity	Value
$S_{x}$	Step length	550 mm
$S_{y}$	Step length	0 mm
$S_{\theta }$	Steering angle	0 degree
*T*	Cycle time	10 s
λ	Duty factor	0.5
*h*	Step height	200 mm

**Table 3:** The attitude regulation results of WT with different $T_{r}$ defined.

Experiment Group	Pitch Angle (Deg)		Roll Angle (Deg)	
	Max. Abs. TE	Std. Deviation	Max. Abs. TE	Std. Deviation
WO	2.945	1.234	3.158	1.298
$T_{r}$ = 2.5 s	1.448	0.567	2.020	0.760
$T_{r}$ = 1.1 s	0.623	0.259	0.821	0.310
$T_{r}$ = 0.8 s	0.671	0.261	0.819	0.333

**Table 4:** The attitude regulation results of different control schemes.

Experiment Group	Pitch Angle (Deg)		Roll Angle (Deg)	
	Max. Abs. TE	Std. Deviation	Max. Abs. TE	Std. Deviation
WO	2.945	1.234	3.158	1.298
WF	1.085	0.464	1.470	0.418
WV	0.818	0.300	1.197	0.325
WT	0.623	0.259	0.821	0.310
WFV	0.604	0.249	0.680	0.203
WFT	0.515	0.169	0.672	0.202

**Table 5:** Walking parameters.

Parameter	Quantity	Value
$S_{x}$	Step length	350 mm
$S_{y}$	Step length	0 mm
$S_{\theta }$	Steering angle	0 degree
*T*	Cycle time	10 s
λ	Duty factor	0.5
*h*	Step height	150 mm

**Table 6:** The attitude regulation results of different control schemes.

Experiment Group	Pitch Angle (Deg)		Roll Angle (Deg)	
	Max. Abs. TE	Std. Deviation	Max. Abs. TE	Std. Deviation
WF	4.127	0.872	2.430	0.769
WV	2.448	0.523	2.086	0.610
WT	1.078	0.436	1.552	0.607
WFV	2.128	0.472	1.056	0.303
WFT	0.914	0.336	0.710	0.273

## References

[1] Bing Z., Meschede C., Chen G., Knoll A., Huang K. (2020). Indirect and direct training of spiking neural networks for end-to-end control of a lane-keeping vehicle. Neural Netw..

[2] Bing Z., Lemke C., Cheng L., Huang K., Knoll A. (2020). Energy-efficient and damage-recovery slithering gait design for a snake-like robot based on reinforcement learning and inverse reinforcement learning. Neural Netw..

[3] Bing Z., Meschede C., Röhrbein F., Huang K., Knoll A. (2018). A Survey of Robotics Control Based on Learning-Inspired Spiking Neural Networks. Front. Neurorobot..

[4] Johnson A.M., Hale M.T., Haynes G.C., Koditschek D.E. Autonomous legged hill and stairwell ascent. Proceedings of the 2011 IEEE International Symposium on Safety, Security, and Rescue Robotics.

[5] Bai L., Hu H., Chen X., Sun Y., Ma C., Zhong Y. (2019). CPG-Based Gait Generation of the Curved-Leg Hexapod Robot with Smooth Gait Transition. Sensors.

[6] Semini C., Barasuol V., Goldsmith J., Frigerio M., Focchi M., Gao Y., Caldwell D.G. (2016). Design of the hydraulically actuated, torque-controlled quadruped robot HyQ2Max. IEEE/ASME Trans. Mechatron..

[7] Gehring C., Coros S., Hutler M., Bellicoso C.D., Heijnen H., Diethelm R., Bloesch M., Fankhauser P., Hwangbo J., Hoepflinger M. (2016). Practice makes perfect: An optimization-based approach to controlling agile motions for a quadruped robot. IEEE Robot. Autom. Mag..

[8] Klein C.A., Olson K.W., Pugh D.R. (1983). Use of force and attitude sensors for locomotion of a legged vehicle over irregular terrain. Int. J. Robot. Res..

[9] Hodoshima R., Doi T., Fukuda Y., Hirose S., Okamoto T., Mori J. (2007). Development of a Quadruped Walking Robot TITAN XI for Steep Slope Operation–Step Over Gait to Avoid Concrete Frames on Steep Slopes. J. Robot. Mechatron..

[10] Irawan A., Nonami K. (2011). Compliant walking control for hydraulic driven hexapod robot on rough terrain. J. Robot. Mechatron..

[11] Zhuang H.C., Gao H.B., Deng Z.Q. (2017). Gait planning research for an electrically driven large-load-ratio six-legged robot. Appl. Sci..

[12] Song S., Waldron K. (1989). Machines That Walk: The Adaptive Suspension Vehicle.

[13] Li Z., Ge Q., Ye W., Yuan P. (2015). Dynamic balance optimization and control of quadruped robot systems with flexible joints. IEEE Trans. Syst. Man Cybern. Syst..

[14] Jiang W.Y., Liu A.M., Howard D. (2004). Optimization of legged robot locomotion by control of foot-force distribution. Trans. Inst. Meas. Control..

[15] Galvez J.A., Estremera J., De Santos P.G. (2003). A new legged-robot configuration for research in force distribution. Mechatronics.

[16] Moosavian S.A.A., Dabiri A. Dynamics and planning for stable motion of a hexapod robot. Proceedings of the 2011 IEEE/ASME International Conference on Advanced Intelligent Mechatronics.

[17] Wang G., Ding L., Gao H., Deng Z., Liu Z., Yu H. (2020). Minimizing the Energy Consumption for a Hexapod Robot Based on Optimal Force Distribution. IEEE Access.

[18] Roy S.S., Choudhury P.S., Pratihar D.K. Dynamic modeling of energy efficient hexapod robot’s locomotion over gradient terrains. Proceedings of the FIRA RoboWorld Congress.

[19] Mahapatra A., Roy S.S., Bhavanibhatla K., Pratihar D.K. Energy-efficient inverse dynamic model of a Hexapod robot. Proceedings of the 2015 International Conference on Robotics, Automation, Control and Embedded Systems (RACE).

[20] Zapolsky S., Drumwright E. Quadratic programming-based inverse dynamics control for legged robots with sticking and slipping frictional contacts. Proceedings of the IEEE/RSJ International Conference of Intelligent Robots and Systems.

[21] Liu Y., Ding L., Gao H., Liu G., Yu H. Efficient force distribution algorithm for hexapod robot walking on uneven terrain. Proceedings of the 2016 IEEE International Conference on Robotics and Biomimetics (ROBIO).

[22] Roy S.S., Pratihar D.K. (2011). Dynamic modeling of energy efficient crab walking of hexapod robot. Appl. Mech. Mater..

[23] Chen C., Guo W., Zheng P., Zha F., Wang X., Jiang Z. Stable Motion Control Scheme Based on Foot-Force Distribution for a Large-Scale Hexapod Robot. Proceedings of the 2019 IEEE 4th International Conference on Advanced Robotics and Mechatronics (ICARM).

[24] Hutter M., Sommer H., Gehring C., Hoepflinger M., Bloesch M., Siegwart R. (2014). Quadrupedal locomotion using hierarchical operational space control. Int. J. Robot. Res..

[25] Yoneda K., Iiyama H., Hirose S. (1994). Sky-Hook Suspension Control of a Quadruped Walking Vehicle. J. Robot. Soc. Jpn..

[26] Huang Q., Fukuhara Y., Chen X. (2007). Posture and vibration control based on virtual suspension model using sliding mode control for six-legged walking robot. J. Syst. Des. Dyn..

[27] Huang Q. (2010). Softly Stable Walk Using Phased Compliance Control with Virtual Force for Multi-Legged Walking Robot. Climbing Walk. Robot..

[28] Wang P.F., Li M.T., Sun L.N. Body Posture Control of Wheeled Foot Quadruped Robot Based on Virtual Suspension Model. Proceedings of the International Conference on Intelligent Robotics and Applications.

[29] Shi Y., Wang P., Wang X., Zha F., Jiang Z., Guo W., Li M. (2018). Bio-inspired equilibrium point control scheme for quadrupedal locomotion. IEEE Trans. Cogn. Dev. Syst..

[30] Gao H., Liu Y., Ding L., Liu G., Yu H. (2019). Low impact force and energy consumption motion planning for hexapod robot with passive compliant ankles. J. Intell. Robot. Syst..

[31] Deng H., Xin G., Zhong G., Mistry M. (2017). Gait and trajectory rolling planning and control of hexapod robots for disaster rescue applications. Robot. Auton. Syst..

[32] Zeng X., Zhang S., Zhang H., Li X., Zhou H., Fu Y. (2019). Leg Trajectory Planning for Quadruped Robots with High-Speed Trot Gait. Appl. Sci..

[33] Erden M.S. (2011). Optimal Protraction of a Biologically Inspired Robot Leg. J. Intell. Robot. Syst..

[34] Garcia E., Gonzalez-De-Santos P. (2010). Using Soft Computing Techniques for Improving Foot Trajectories in Walking Machines. J. Robot. Syst..

[35] Zha F., Chen C., Guo W., Zheng P., Shi J. (2019). A free gait controller designed for a heavy load hexapod robot. Adv. Mech. Eng..

[36] Dong Y., Guo W., Zha F., Liu Y., Chen C., Sun L. (2020). A Vision-Based Two-Stage Framework for Inferring Physical Properties of the Terrain. Appl. Sci..

